# Biological Diversity, Ecological Health and Condition of Aquatic Assemblages at National Wildlife Refuges in Southern Indiana, USA

**DOI:** 10.3897/BDJ.3.e4300

**Published:** 2015-01-12

**Authors:** Thomas P. Simon, Charles C. Morris, Joseph R. Robb, William McCoy

**Affiliations:** † Indiana University, Bloomington, IN 46403, United States of America; ‡ US National Park Service, Indiana Dunes National Lakeshore, Porter, IN 47468, United States of America; § US Fish and Wildlife Service, Big Oaks National Wildlife Refuge, Madison, IN 47250, United States of America; | US Fish and Wildlife Service, Patoka River National Wildlife Refuge, Oakland City, IN 47660, United States of America

**Keywords:** Distribution, Conservation, Ecological health, Fish, Crayfish, Macroinvertebrates

## Abstract

The National Wildlife Refuge system is a vital resource for the protection and conservation of biodiversity and biological integrity in the United States. Surveys were conducted to determine the spatial and temporal patterns of fish, macroinvertebrate, and crayfish populations in two watersheds that encompass three refuges in southern Indiana. The Patoka River National Wildlife Refuge had the highest number of aquatic species with 355 macroinvertebrate taxa, six crayfish species, and 82 fish species, while the Big Oaks National Wildlife Refuge had 163 macroinvertebrate taxa, seven crayfish species, and 37 fish species. The Muscatatuck National Wildlife Refuge had the lowest diversity of macroinvertebrates with 96 taxa and six crayfish species, while possessing the second highest fish species richness with 51 species. Habitat quality was highest in the Muscatatuck River drainage with increased amounts of forested habitats compared to the Patoka River drainage. Biological integrity of the three refuges ranked the Patoka NWR as the lowest biological integrity (mean IBI reach scores = 35 IBI points), while Big Oaks had the highest biological integrity (mean IBI reach score = 41 IBI points). The Muscatatuck NWR had a mean IBI reach score of 31 during June, which seasonally increased to a mean of 40 IBI points during summer. Watershed IBI scores and habitat condition were highest in the Big Oaks NWR.

## Introduction

The National Wildlife Refuge system is an invaluable resource in the protection of biological diversity (Loomis and White 1996; Meretsky et al. 2006; Glicksman and Cumming 2012). The conservation of imperiled species and the protection of biological integrity at national wildlife refuges are mandates of the Federal Fish and Wildlife Service (Policy 601 FW 3). This policy considers the protection of a broad spectrum of fish, wildlife, and habitat resources and evaluates processes necessary to restore lost or severely degraded components. Ecosystem services provided by refuges are assessed at a total value of US $32.3 billion/year (Ingraham and Foster 2008).

National Wildlife Refuge System Administration Act of 1966 as amended by the National Wildlife Refuge System Improvement Act of 1997, 16 U.S.C. 668dd-668ee (Refuge Administration Act), Section 4(a)(4)(B) states that "In administering the System, the Secretary shall... ensure that the biological integrity, diversity, and environmental health of the System are maintained for the benefit of present and future generations of Americans" (United States Fish and Wildlife Service 1999). Biological integrity, diversity, and environmental health can be described at various landscape scales from refuge to ecosystem, national, and international levels (Davis and Simon 1995, Simon 2000, Noss 2004, Fischman 2005). Each landscape scale has a measure of biological integrity, diversity, and environmental health that depends on existing habitats, ecosystem processes, and alterations (Simon 2000, Morris et al. 2005). Biological integrity, diversity, and environmental health can vary among refuges and often within refuges over time based on system resistance and resilience (Carpenter and Brock 2004).

The goal of biological integrity, unlike fishable and swimmable goals, encompasses all factors affecting the ecosystem (Simon 2000). Karr and Dudley (1981) define biological integrity as “the capability of supporting and maintaining a balanced, integrated, adaptive community of organisms having a species composition, diversity, and functional organization comparable to that of the natural habitat of the region.” That is, a site with high biological integrity will have had little or no influence from human society. Biological integrity lies along a continuum from a system extensively altered by significant human impacts in the landscape to a natural system undisturbed by anthropogenic influences on the system (Hughes 1995, Simon 2000, Stoddard et al. 2006). No landscape retains absolute biological integrity, diversity, and environmental health; however, the prevention of further loss of natural biological features and processes is a protection mandate.

Biological diversity is evaluated at various taxonomic levels, and for purposes of Endangered Species Act implementation at distinct population segments (Waples 1991, United States Fish and Wildlife Service and National Marine Fisheries Service 1996). Evaluations of biological diversity begin with population surveys and studies of species level flora and fauna, which are the basic elements of biodiversity. The refuge system's focus is on native species and natural communities, such as those found under historic conditions. Biological diversity is evaluated at various landscape scales, while evaluations of biological diversity focus is at the refuge scale (Meretsky et al. 2006). The maintenance of populations of breeding individuals that are genetically viable and functional require necessary provision for the breeding, migrating, and wintering needs of species. Every effort is made to maximize the size of habitat blocks and maintain connectivity between blocks of habitats, unless such connectivity causes adverse effects on wildlife or habitat, such as by facilitating the spread of invasive species (Fischman 2005).

Ecological health is defined by the U.S. Fish and Wildlife Service Refuge System as the extent that environmental composition, structure, and function have been altered from historic conditions (United States Fish and Wildlife Service 1999). Environmental composition refers to abiotic components such as air, water, and soils that are integrated with biotic components. Environmental structure refers to the organization of abiotic components, such as atmospheric layering, aquifer structure, and topography. Environmental function includes the abiotic processes, such as wind, tidal regimes, evaporation, and erosion. A diversity of heterogeneous abiotic composition, structure, and function supports a variety of biological composition, structure, and function.

Limited biological integrity studies have been conducted on National Wildlife Refuges, with the exception of contaminant studies at the Patoka River National Wildlife Refuge (Simon et al. 1995, Simon 2004, Simon and Thoma 2003, Simon and Thoma 2003, Simon and Thoma 2006), a baseline survey at the Big Oaks National Wildlife Refuge (Pruitt et al. 1994), and a contaminants investigation at Patoka River, Muscatatuck, and Big Oaks NWR (Simon 2008). The current study documents an inventory of aquatic assemblage biodiversity from three National Wildlife Refuges in southern Indiana and evaluates the status and condition of refuge biological integrity based on indices of biotic integrity and biological diversity indices compared to historical biodiversity information.

## Materials and methods

**Study area**

**Figure 1.**
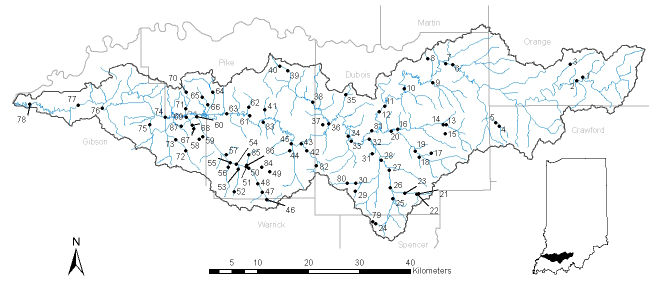
Distribution of sites sampled during an investigation of the Patoka River drainage. Numbers refer to site location in Supplemental materials Appendix a (Suppl. material 1).

The Patoka River watershed (Fig. 1) has a drainage basin of approximately 2170.4 km^2^ (838 mi^2^) and has a wide range of known conditions that are impairing NWR stream quality including acid mine drainage, oil and gas exploration, and impacts from coal mining (Simon et al. 1995). The area supports the largest known populations of the Indiana crayfish (*Orconectes indianensis* Hay 1896), a former Federal candidate species (Simon and Thoma 2006). Numerous stream segments are listed by the State of Indiana as “not meeting” aquatic life designated uses because of metal contamination (Simon and Thoma 2003, Simon 2008). The entire length of the South Fork Patoka River is listed as “not meeting” aquatic life designated uses because of acid mine drainage contamination.

**Figure 2.**
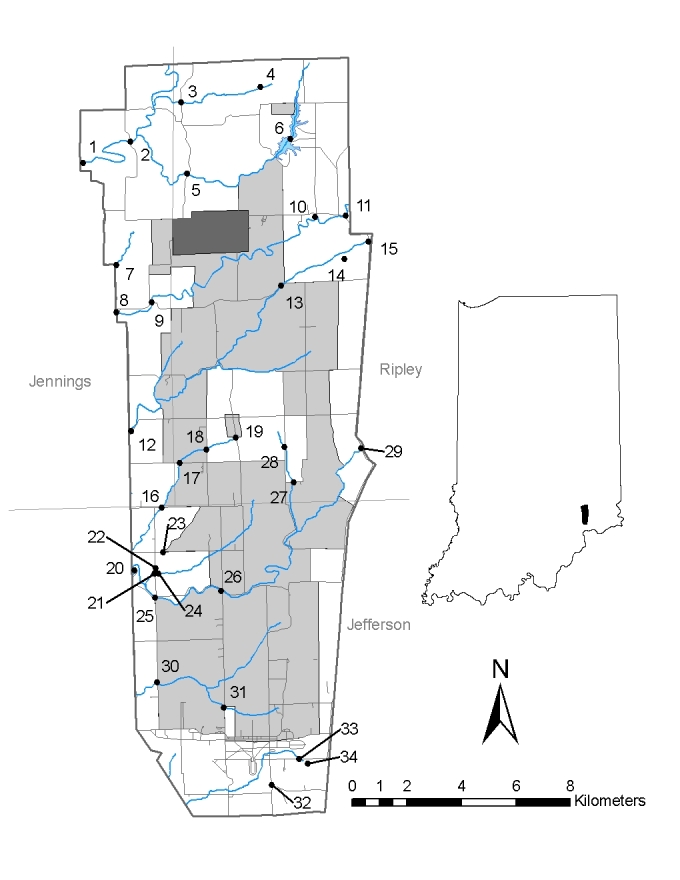
Distribution of sites sampled during an investigation of the Big Oaks National Wildlife Refuge. Numbers refer to site locations in Supplemental materials Appendix b (Suppl. material 1).

**Figure 3.**
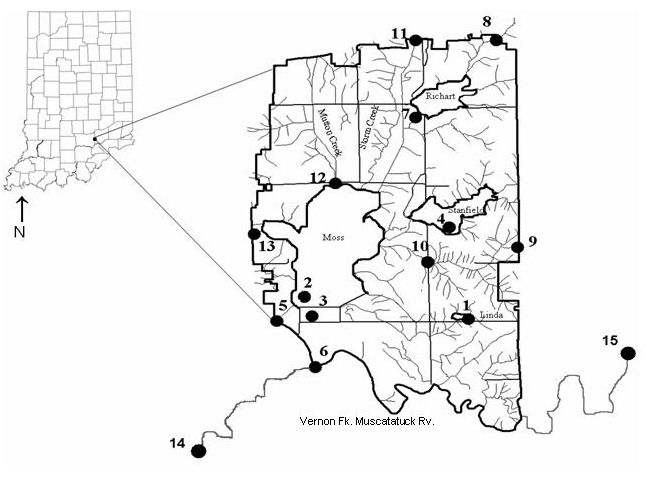
Distribution of sites sampled in the Muscatatuck NWR within Jennings and Jackson Counties in southcentral Indiana. Black dots denote sample locations and numbers correspond to site numbers in Supplemental materials Appendix c (Suppl. material 1).

Sampling was conducted over two years in both the Patoka and Vernon Fork of the Muscatatuck river drainage. The Patoka River National Wildlife Refuge (NWR) is the sole refuge in the Patoka watershed (Fig. 1), while two refuges occur in the Vernon Fork of the Muscatatuck River, including the Big Oaks (Fig. 2) and Muscatatuck (Fig. 3) NWRs. The Muscatatuck River includes a 2952.6 km2 (1,140 mi^2^) watershed that includes a wide range of biological habitats and environmental conditions (Figs 2, 3). The Vernon Fork of the Muscatatuck watershed includes Sloan’s crayfish (*Orconectes sloanii* Bundy 1876), a species of special interest to US Fish and Wildlife Service, Region 3, which has experienced intensive invasion threats from the rusty crayfish (*Orconectes rusticus* Girard 1852). Nutrient impacts are pervasive throughout the Vernon Fork watershed. The Muscatatuck NWR receives runoff drainage through Sandy Branch and Mutton Creek from the City of Seymour, Indiana, and from high density residential land uses. Metal levels in fish tissue exceeded fish consumption advisories within several of the lakes on the Muscatatuck NWR (United States Fish and Wildlife Service 2003). The State of Indiana determined that several streams entering the Muscatatuck NWR are listed as “not meeting” designated uses for aquatic life. Perhaps a larger landscape issue is present at Big Oaks NWR, which incorporates portions of Jefferson Proving Ground, a former military base that has documented impairments from exploded ordnance, depleted uranium, and metal contamination (Simon 2008).

**Study design**

Sampling design incorporated a random probability selection for a portion of sites based on bridge access. Contamination at Big Oaks NWR required crews to access streams from bridge access points due to safety concerns from unexploded ordinance. As a result, all of the random sampling was conducted at bridge access points to maintain consistency. A panel survey design stratified sites with some selected from prior surveys (Simon et al. 1995, Simon et al. 2005a), while a subset of random probability sites was sampled for aquatic macroinvertebrates and crayfish assemblages (see Suppl. material 1 for list of sites). Fifty probability sites and 37 targeted sites were sampled on the Patoka River NWR (Fig. 1), 30 probability and 4 targeted sites were sampled on the Big Oaks National Wildlife Refuge in Jennings, Jefferson, and Ripley counties (Fig. 2), and 20 probability and a single targeted site selected at the Muscatatuck NWR in Jackson and Jennings counties (Fig. 3). These sites represent a variety of habitat types including lakes, ponds, wetlands, streams, and large rivers (Suppl. material 1).

Sampling gear was selected for each of the appropriate habitat types. Lake and wetland areas were sampled using a boat mounted Smith Root 2500 watt DC generator unit. Large to medium size streams (> 8 m wetted width) were assessed using a long-line or backpack electrofishing unit. Small streams (< 8 m wetted width) were assessed using a Smith-Root DC generator backpack unit. Sampling of streams was conducted along a linear reach based on 15 times the wetted stream width (Leopold et al. 1964). Sample reach distance length increased with stream width so that a minimum of 50 m (wetted width <3.3) and maximum of 500 m was sampled. Lakes, ponds, wetlands and moist soil units (MSU) reaches were sampled based on 500 m linear distance and 1800 s. Lake reaches were selected based on natural shoreline features, which included intact riparian vegetation and bank condition. Two 500 m reaches were sampled on Lake Linda, Stansfield Lake, Moss Lake, and MSU with reaches distributed on opposing shores. Due to shallow conditions in Moss Lake, about 500 m of accessible water was sampled at a single site.

**Field and Laboratory methods**

*Fish collection methods*. Daytime inventories were conducted using standard fish community sampling equipment during the summer season (i.e., June-September). The appropriate sampling gear for each site was determined by the field crew chief. Sampling was conducted by the same crew leader, using the same techniques, and equipment, during all sampling periods. Five percent of sites were sampled by both crew leaders to validate crew performance. No statistical difference (Student’s t-test, α = 0.05) was observed in results between crews. Relative abundance (catch per unit effort or CPUE is the number of fish per minute of electrofishing effort) data were gathered by performing surveys at reaches using appropriate electrofishing gear. Sampling gear included a model 6A Smith-Root boat-mounted electrofishing unit in nonwadeable, large main stem rivers and lakes, while Smith Root backpack and longline systems were used in tributaries. Longline units used the same generator and transformer unit, i.e., 3500 watt DC generator and 6A Smith Root unit, as the boat mounted unit. Gear selection was based on stream width with longline units used on large, wadeable wetted widths (>8 m).

Electrofishing surveys included systematic sampling of all representative habitats within each reach, including shallows, instream cover, and the thalweg or deepest point in the cross sectional profile. A representative sample was collected from each reach. Captured fish were placed into a live well until a sampling event was completed. Each survey event included documentation of species identification, batch weight, number of fish captured, presence of external disease including deformities, eroded fins, lesions, and tumor anomalies (DELTs) for each individual and an estimate of qualitative habitat condition (Rankin 1995).

Fish identified in the field had vouchers of 2-3 individuals for later taxonomic verification, while difficult species and other small minnow, darter, and madtom species were preserved in 10% formalin for laboratory processing using standard taxonomic keys (Gerking 1955, Smith 1979, Trautman 1981, Simon et al. 1992, Simon 2011). Scientific names are included in Tables 1, 2, 5 including authorities in Table 1.

*Macroinvertebrate collection methods*. Daytime macroinvertebrate assemblages were sampled using a “representative habitat sampling” procedure developed for streams (Simon and Stewart 1998). D-nets were used to collect 20-efforts within representative habitat throughout the reach distance length. An effort is a 60-second sample of a specific habitat type that represents a proportion of the reach scale habitat. Efforts were established to correlate with the predominant habitats present within the reach. For example, habitats were segregated into rock, fines, overhanging vegetation, woody debris, coarse particulate material, and other categories (Simon and Stewart 1998). So, if rocky riffle habitat represents 50% of the habitat within the stream reach, then 10 of the 20 efforts would be collected within that particular habitat type. The individuals collected during the 20 effort D-net sampling were composited and preserved in 95% ethanol for laboratory sorting.

Samples were brought to the laboratory for sorting of stream bank composited samples. The sample contents were placed into a 250 mm x 250 mm (10 x 10 inch) gridded sorting pan (Simon and Stewart 1998). Sorting was done until 300 organism subsample was obtained. The grid picked was selected using a random number generator (i.e., Research Randomizer http://www.randomizer.org/form.htm) to determine the appropriate square to be sorted. Sorting included the entire square until the 300th organism was picked; however, the square that contained the 300^th^ individual was sorted until it was fully picked. Reach macroinvertebrate density varied with sample and ranged from a maximum of all 100 squares picked (and either less than 300 individuals or more than 300 individuals) to fewer than all squares sorted and less than 300 total individual organisms.

After the completion of the 300 organism sort, a 15 minute large-rare examination was completed for samples that were not entirely picked. A large-rare sort included the remaining squares were scanned for taxa that had not been previously observed with emphasis on unique taxa not previously observed. Individuals from the large-rare pan sorted pick were identified and data content was incorporated into species richness metric calculations, but were not included in trophic or relative abundance metrics following standard procedures (Simon and Stewart 1998). All individuals were identified to the lowest possible taxonomic levels, i.e., genus or species, following state-of-the-art taxonomic resolution appropriate for that particular taxon (Pecarsky et al. 1990, Smith 2001, Merritt et al. 2008). Scientific names including authorities are listed in Table 3.

*Crayfish collection methods*. Crayfish sampling included the evaluation of primary, secondary, and tertiary burrowing species (Simon 2001, Simon 2004). Primary, secondary, and tertiary burrowing as defined by Hobbs (1981). Primary burrowers are terrestrial species that do not require submergence under water and dig extensive burrows with complex ventilation holes. Secondary burrowers dig burrows into the side of the bank and inhabit both aquatic and terrestrial habitats, while tertiary burrowers are mostly aquatic and do not dig burrows other than shallow depressions in stream beds as streams desiccate.

Catch-per-unit-effort (CPUE) is based on the number of individuals collected per effort required to sample each site based on the stream size. Greater sized streams had more effort. Effort is based on the 15 times the wetted width in a linear distance sampled.

Burrowing crayfish were collected using excavation and plunging techniques (Simon 2001, Simon 2004). Individuals were coaxed from their burrow by pouring water down the burrow and agitating the water. If the crayfish failed to emerge, then a toilet plunger was used to force the crayfish from the burrow (Simon 2001). If that failed to dislodge the crayfish, then a hand shovel was used to excavate the burrow and retrieve the individual (Simon 2004). Secondary and tertiary burrowers were collected using a backpack electrofishing unit. Secondary burrowers were also collected by hand by turning over large rocks in the stream. By flipping rocks, the crayfish could be easily collected by hand. Tertiary burrowers were collected with a dip net or by hand. All crayfish species collected from each site had an estimate of relative abundance based on a standard catch-per-unit-effort per site (Simon 2004) or number of individuals per square meter sampled.

Specimens were preserved in 70% ethanol, returned to the laboratory for processing, and identified using standard taxonomic references including Page (1985), Hobbs (1989), and Taylor and Schuster (2004). Scientific names and authorities are cited in Table 4.

**Assessment of Biological Integrity**

Simon and Dufour (1998) developed and calibrated an index of biotic integrity (IBI) for fish assemblages in the Eastern Corn Belt Plain Ecoregion based on data from 200 least impacted sites. Reference condition models are a conservative approach for establishing expected attributes of the biological assemblage, since these models recognize that pristine conditions are limited or in such small distributions that they might not be extant (Hughes 1995). Simon and Dufour (1998) developed reference condition calibrations following Karr et al. (1986) and used a maximum species area curve to determine expectations and scoring criteria (Fausch et al. 1984). Scoring classifications and expectations follow Karr (1981).

Rankin (1995) created a habitat condition index based on substrate, riparian corridor, stream sinuosity, cover, riffle-run quality, pool quality, and habitat cycle percentage and gradient. The Qualitative Habitat Evaluation Index (QHEI) is a measure of condition that was originally developed in the Midwestern United States. Scores range from 0-100 ponts with higher values representing increasing habitat quality. Habitat condition scores greater than 66 points are considered meeting aquatic life designated uses. Rankin (1995) found that habitat condition increased directly and proportionally with fish assemblage quality.

**Statistical analyses**

Cumulative frequency distributions of IBI scores and descriptive statistics for each refuge were completed using Statistica (Statsoft 2007). Relative abundance was based on the number of individuals per unit distance and time (Environmental Protection Agency 1988) and was transformed into percent occurrence based on total numbers of individuals collected at each site. This approach assures that species richness area curves are comparable between watersheds and ecoregions. Spline-smoothed pleths were created based on reach scale average species richness and then averages were joined to produce hot-spot biodiversity, habitat condition and index of biotic integrity depictions for each watershed (Morris et al. 2005).

## Taxon treatments

### Synurella dentata

Hubricht 1943

#### Distribution

Patoka River NWR: 22, 27, 29, 41, 64-66, 82, 83

Muscatatuck NWR: 8-11, 13, 19, 20

Big Oaks NWR: 29

The toothed spring amphipod (*Synurella dentata*) was collected by Lewis and Rafail (2002)) from Big Creek from Three Raiders Monument. This species is ubiquitous in springs and some caves.

#### Ecology

*Synurella dentata* was a dominant species represented by a 16% occurrence and is considered to have a wide habitat tolerance. The toothed spring amphipod (*Synurella dentata*) is a cave spring species associated with karst habitats (Lewis and Rafail 2002).

#### Conservation

The toothed spring amphipod has a species conservation rank of S4/G5.

### Lirceus fontinalis

Rafinesque-Schmaltz 1820

#### Distribution

Patoka River NWR: 1, 3, 22, 25, 27, 29-31, 35, 36, 73, 79

Muscatatuck NWR: 7-13, 16, 19, 20

Big Oaks NWR: 13, 15, 20

The species ranges from southern Indiana, Kentucky, southwestern Ohio, and northern Tennessee (Lewis and Rafail 2002). The bluegrass spring isopod was collected from caves in the Middle Fork, Big Creek, and Graham Creek watersheds.

#### Ecology

*Lirceus fontinalis* (24%) was dominant within the refuges and is considered to have a wide habitat tolerance. The bluegrass spring isopod is a cave spring species associated with karst habitats (Lewis and Rafail 2002).

#### Conservation

The bluegrass spring isopod has a conservation species rank is S3/G4.

### Orconectes (Faxonius) indianensis

(Hay 1896)

#### Distribution

Patoka River NWR: 1-3, 6-7, 17, 19, 22, 25, 26, 29, 34, 38-40, 63

#### Ecology

An additional sixteen records of Indiana crayfish were found from areas surrounding the refuge. These sites included solid rock substrate habitats as described by Simon and Thoma (2003) and Simon and Thoma (2006).

#### Conservation

This former Federal candidate species does not warrant protection based on current and previously known collection information (Simon and Thoma 2003, Simon and Thoma 2006). The Patoka River supports the largest known populations of the Indiana crayfish, which is a former Federal candidate species (Simon and Thoma 2006).

### Orconectes (Rhoadesius) sloanii

(Bundy 1876)

#### Distribution

Muscatatuck NWR: 5, 6, 10, 13-15, 20

Big Oaks NWR: 5, 7-13, 15, 16, 20, 23, 25, 27, 29, 30, 32

Extensive survey of southwestern Ohio and southeastern Indiana documented the distribution and status of Sloan’s crayfish. Closer inspection of St John's (1988) distribution maps show that areas included within the Big Oaks National Wildlife Refuge were represented by only Sloan’s crayfish and did not possess the invasive rusty crayfish. Our sampling results found similar results as St. John (1988).

#### Ecology

Sloan’s crayfish was collected at 76.5% of the sites in the Big Oaks refuge. Relative abundance averaged 13.6 individuals per site. Mean density of Sloan’s crayfish was 0.272 individuals per square meter. Sloan’s crayfish was collected at 36.8% of the sites on the Muscatatuck refuge. Relative abundance averaged 9.14 individuals per site. Mean density of Sloan’s crayfish was 0.182 individuals per square meter.

#### Conservation

Sloan’s crayfish (*Orconectes sloanii*) is a species of special interest to US Fish and Wildlife Service, Region 3, which has experienced intensive invasion threats from the rusty crayfish (*Orconectes rusticus* Girard 1852). Nutrient impacts are pervasive throughout the Vernon Fork watershed. The Muscatatuck NWR receives runoff drainage through Sandy Branch and Mutton Creek from the City of Seymour, Indiana, and from high density residential land uses.

Sloan’s crayfish is stable and has a relatively high relative abundance in the Big Oaks and Muscatatuck National Wildlife Refuges. No instances of rusty crayfish were observed in either of the refuges (Table 4).

### Notropis ariommus

(Cope 1867)

#### Distribution

Big Oaks NWR: 1, 11, 20

The collection of popeye shiner represent the first record for this species in Indiana since the species was originally described from the White River near Indianapolis in the late 1800’s. During this study specimens were collected from Otter Creek, Big Graham Creek, and Big Creek (Table 5).

#### Ecology

The species was collected from moderate sized flowing rivers over cobble and gravel substrates.

#### Conservation

The species has been considered extirpated within Indiana, but with these records should be considered for additional study to determine the species current status.

### Centrarchus macropterus

(Lacepede 1801)

#### Distribution

Patoka River NWR: 62, 68

Muscatatuck NWR: 2, 5, 12

Flier is a centarchid species largely associated with the southeastern and eastern United States. Its distribution is restricted to the Coastal Plain from the Chesapeake Bay to Eastern Texas and north through the Mississippi Embayment to southern Illinois and Indiana (Smith 1979, Lee et al. 1980). Records for Indiana depict its distribution to be limited to the southwestern and central portions of the state (Gerking 1945). The flier was collected from three sites in this study including the Vernon Fork, from Mutton Creek, and from Moss Lake (Table 1). These records constitute the furthest northern and easternmost collections within the species range (Gerking 1945, Lee et al. 1980).

#### Ecology

A total of 11 individuals were collected from the Muscatatuck NWR. Our flier individuals occurred in pool and low-flow, basic gradient streams with wood debris (Gerking 1945, Lee et al. 1980).

### Lepomis (Lepomis) symmetricus

Forbes 1883

#### Distribution

Patoka River NWR: 52

The Bantam sunfish is reported from the Patoka River watershed from Rough Creek. This watershed has experienced extensive acid mine drainage impacts.

#### Ecology

*Lepomis symmetricus* was collected from a pool about 1 m in depth from the areas upstream from the bridge.

#### Conservation

*Lepomis symmetricus* is rare and is considered endangered within the State of Indiana.

### Ammocrypta (Ammocrypta) pellucida

(Putnam 1863)

#### Distribution

Muscatatuck NWR: 15

#### Ecology

The eastern sand darter (*Ammocrypta pellucida*) was collected from one site on the Vernon Fork Muscatatuck River (Table 1). Three individuals were collected from the Vernon Fork over shallow, sandy-riffle habitat.

#### Conservation

The eastern sand darter was once recognized as state threatened species based on limited presence in the state (Simon et al. 1992), but has since been removed from threatened status. The eastern sand darter is still considered rare and is susceptible to impacts of habitat degradation (Simon 1993).

### Etheostoma (Etheostoma) histrio

Jordan and Gilbert 1887

#### Distribution

Patoka River NWR: 78

Muscatatuck NWR: 6, 14

The harlequin darter was thought to be extripated from Indiana until its rediscovery within the White River Drainage in 1991 (Simon and Kiley 1993). It has since been collected from other subwatersheds within the White River and also from the Patoka River (Simon et al. 1995). The harlequin darter was collected from Vernon Fork Muscatatuck River at two sites, while the rediscovery of it was documented in the mainstem Patoka River upstream to the mouth of South Fork Patoka River. These records constitute the furthest removed records for the harlequin darter from the main stem of either branch of the White River (Table 1).

#### Ecology

Two Harlequin darter individuals were collected over gravel/sand riffles with swift current.

## Analysis

**Habitat patterns at a watershed scale**

**Figure 4.**
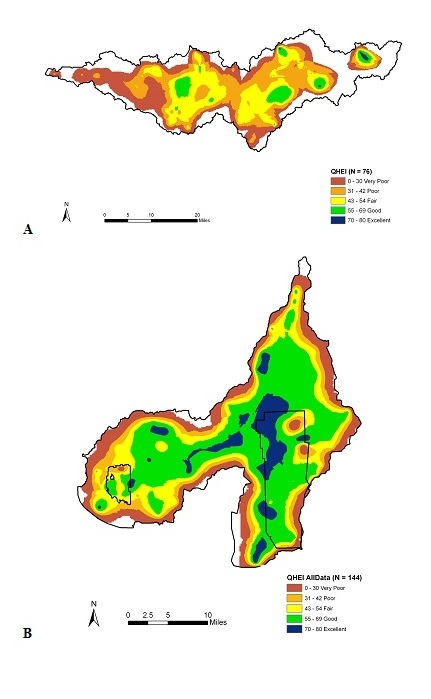
Watershed scale patterns in habitat condition for the Patoka River (A) and Muscatatuck River (B) drainages using spline smoothed joined mean scores based on the Qualitative Habitat Evaluation Index (QHEI). Position and outline of the three National Wildlife Refuges are shown in relationship to the watershed (Suppl. materials 2, 3, 4).

There is a direct correlation between landscape scale ecological patterns and reach scale habitat measures (Burcher et al. 2008). Natural landscapes provide important ecological services that promote biological diversity and integrity. The QHEI scores at a watershed scale show that the Patoka River drainage (Fig. 4a) has the lowest overall habitat scores compared to the Muscatatuck River (Fig. 4b). Patoka River drainage habitat is highest in the upper watershed surrounding Hoosier National Forest and Patoka Lake. The lowest habitat scores were associated with the declining habitat condition in the lower watershed. This decline is attributed to agriculture, legacy mining, channel modification, and oil and gas exploration (Fig. 4a).

The Muscatatuck River drainage comparatively has the highest habitat quality of the two drainages. The Muscatatuck NWR has the lower habitat quality associated with the refuge borders compared to the Big Oaks NWR (Fig. 4b). Edge effects from agricultural land use was the primary factor effecting the eastern boundary of the Big Oaks refuge, while at the Muscatatuck NWR the streams drain from the north to south in orientation. The northern edge of the Muscatatuck refuge is most influenced by agriculture, while the eastern and western margins of the refuge had the highest habitat scores.

**Biological diversity, composition, and assemblage changes**

**Patoka River NWR**

*Species richness and composition*. We collected 9,658 individuals representing 82 fish species from streams and rivers on the Patoka River National Wildlife Refuge and tributaries (Table 1 and Table 2). Dominant families included the Cyprinidae (20 species), Centrarchidae (15 species) and Catostomidae (12 species), which was comparable to similar historical catches (Simon et al. 1995, Simon 2005, Simon et al. 2005a). Dominant species included longear sunfish (*Lepomis megalotis*)(1,314 individuals), central stoneroller (*Campostoma anomalum*) (827 individuals), bluegill (*Lepomis macrochirus*)(461 individuals) and striped shiner (*Luxilus chrysocephalus*) (378 individuals). These species were dominant in pool habitats (longear sunfish and bluegill), headwater streams (central stoneroller), and in wadable stream pool habitats (striped shiner) draining the refuge (Table 2). During the second sampling season four dominant species included creek chub (*Semotilus atromaculatus*) (671 individuals), western mosquitofish (*Gambusia affinis affinis*)(568 individuals), bluntnose minnow (*Pimephales notatus*) (524 individuals), and blackstripe topminnow (*Fundulus notatus*)(423 individuals). These species are tolerant forms that can occupy acid mine drainage streams (pH < 5).

*Macroinvertebrate species richness and composition*. No previous macroinvertebrate investigation has been conducted in the Patoka River drainage at the lowest taxonomic resolution levels. During this investigation of the Patoka River watershed, 355 taxa representing 93 families were collected (Table 3). Dominant orders included the Hemiptera and Diptera (12 families), Coleoptera (11 families), and Ephemeroptera (8 families). Among the most diverse macroinvertebrate taxa was the Diptera or flies and midges (103 taxa), Hemiptera or true bugs (67 taxa), and the Odonata or dragonflies (47 taxa) (Table 3). Based on comparing macroinvertebrate species composition differences between annual events, 2006 surveys found 16 mayfly taxa compared to 11 mayfly taxa in 2007. Unique taxa collected during 2006 included, *Baetis intercalaris*, *Procloeon* spp., *Psuedocloeon* spp., *Hexagenia limbata*, *Tricorythodes* spp., and *Choroterpes* spp., while during 2007 *Ameletus* spp., *Acerpenna pygmaea*, *Plauditus dubius*, *Plauditus* spp., and *Nixes* spp. were collected (Table 3). Three stonefly taxa were collected during the inventory including *Acroneuria* spp. and *Neoperla* spp during 2006 and *Isoperla* spp. during 2007 (Table 3). Caddisfly taxa collected during 2006 and 2007 included 25 taxa (Table 3). During 2006, 13 caddisfly taxa were unique including, *Hydropsyche betteni-depravata, Hydropsyche cuanis, H. hageni, H. simulans, Oxyethira* spp., *Nectopsyche candida*, *N. exquisite*, *Nectopsyche* spp., *Oecetis cinerascens*, *Chimarra aterrima*, *C. obscura*, *Cernotina spicata*, and *Neurclipsis crepuscularis*. During 2007, seven taxa were unique including, *Hydropsyche betteni*, *Ironoquia* spp., *Chimarra* spp., *Ptilostomis* spp., *Polycentropus* spp., *Rhacophila* spp., and *Neophylax* spp.

*Crayfish species richness and composition*. Several studies of crayfish near the Patoka River NWR have been conducted (Simon and Thoma 2003, Simon et al. 2005a, Simon and Thoma 2006, Simon and Morris 2009). Simon and Thoma (2003) described the crayfish assemblages of the Patoka River watershed including species occurring around the National Wildlife Refuge. A new species of crayfish, the paintedhand mudbug *Cambarus polychromatus* Thoma, Jezerinac, and Simon 2005 was described from Flat Creek on the Patoka River NWR (Thoma et al. 2005). Simon and Thoma (2006) described the onservation status of the Indiana crayfish, while Simon et al. (2005b) described the reproductive biology, distribution, and habitat needs of species occurring in the Patoka River drainage. Simon and Morris (2009) studied the effects of oil brine and acid mine leachate on the crayfish fauna of the Patoka River watershed. The current study evaluated 88 sites (Suppl. material 1), which include the same locations as previously sampled for fish and macroinvertebrate assemblages (Table 4). Similar species richness in the area surrounding the Patoka River NWR was found as in previous studies (Simon and Thoma 2003). The dominant species was the calico crayfish (*Orconectes immunis*), which was found throughout the refuge and areas surrounding the Patoka River NWR. Both northern crayfish (*Orconectes virilis*) and White River crayfish (*Procambarus acutus*) were collected from single locations. Two species of primary burrowing crayfish were collected from the refuge (Table 4). The paintedhand mudbug was more common than the Great Plains mudbug. No invasive crayfish species were found during the present sampling in the Patoka River watershed.

**Changes in Biological diversity**

*Fish assemblage record changes*. Five times as much collection effort was expended in the Patoka watershed since 1992 than had previously occurred over the last two centuries. Simon et al. (1995) documented the increase in species diversity as a result of increased sampling intensity. Nine first species records were found in the watershed between 1992-2002, including threadfin shad (*Dorosoma petenense)*, cypress minnow (*Hybognathus hayi*), ribbon shiner (*Lythrurus fumeus*), pallid shiner (*Notropis amnis*), Southern redbelly dace (*Chrosomus erythrogaster*), fathead minnow (*Pimephales promelas*), blacknose dace (*Rhinichthys obtusus*), and starhead topminnow (*Fundulus dispar*). The range extension of ribbon shiner may have been a result of misidentification since prior identification of redfin shiner (*Lythrurus umbratilis*) was documented. This species does not occur in the upper portion of the Patoka watershed. The ribbon shiner was previously known from only a few small streams in southwestern Indiana (Simon 2011). Bait-bucket release of fathead minnow into the watershed was speculated by Simon et al. (1995), while the presence of threadfin shad was probably a result of immigration from upstream reservoir habitats. Pallid shiner and starhead topminnow presence suggest that water quality conditions were improving. These fish species are considered sensitive to acidity and turbidity (Simon and Dufour 1998). Newly found species since 1993 added the discovery of lake chubsucker (*Erimyzon sucetta*) and paddlefish (*Polyodon spathula*) (Doug Carnahan, Indiana DNR, personal communication) in oxbow lakes from the floodplain wetlands. Other new discoveries include goldeye (*Hiodon alosoides*), shoal chub (*Macrhybopsis hyostoma*), channel shiner (*Notropis wickliffi*), silver redhorse (*Moxostoma anisurum*), and stonecat (*Noturus flavus*). Species that were rediscovered include river shiner (*Notropis blennius*), sand shiner (*N. stramineus*), bullhead minnow (*Pimephales vigilax*), shorthead redhorse (*Moxostoma macrolepidotum*), brook silverside (*Labidesthes sicculus*), yellow bass (*Morone mississippiensis*), and smallmouth bass (*Micropterus dolomieu*).

The increase in the number of species records was directly a result of increased sampling effort. The rediscovery of lake chubsucker, paddlefish, rock bass (*Ambloplites rupestris*), slough darter (*Etheostoma gracile*), harlequin darter, blackside darter (*Percina maculata*), dusky darter, and banded sculpin (*Cottus carolinae*) are all species sensitive to siltation and acidity. The increased occurrence of these species in the watershed may be an environmental indicator of recovery. Species composition additions during 2006 included Spotted gar (*Lepisosteus oculatus*), longnose gar (*L. osseus*), skipjack herring (*Alosa chrysochloris*), ribbon shiner, southern redbelly dace, white sucker (*Catostomus commersonii*), black buffalo (*Ictiobus niger*), spotted sucker (*Minytrema melanops*), black redhorse (*Moxostoma duquesnei*), stonecat, banded sculpin, rock bass, smallmouth bass, white crappie (*Pomoxis annularis*), black crappie (*P. nigromaculatus*), harlequin darter, and slenderhead darter (*Percina phoxocephala*). Many species, such as rock bass, southern redbelly dace, banded sculpin, smallmouth bass, and slenderhead darter, were collected in areas upstream of the refuge in high gradient tributaries that drain the Hoosier National Forest. While large river, floodplain species such as spotted and longnose gar, black buffalo, spotted sucker, and harlequin darter were collected from the Patoka River downstream of the refuge. Forty-eight of the 82 fish species (58.5%) collected during 2006 were also collected during 2007. During 2007, first records for species composition additions included goldeye, threadfin shad, bigeye chub (*Hybopsis amblops*), bighead carp (*Hypophthalmichthys nobilis*), bullhead minnow, quillback (*Carpiodes cyprinus*), lake chubsucker, shorthead redhorse (*Moxostoma macrolepidotum*), white catfish, tadpole madtom (*Noturus gyrinus*), brindled madtom (*Noturus miurus*), freckled madtom (*Noturus nocturnus*), central mudminnow (*Umbra limi*), flier (*Centrarchus macropterus*), orangespotted sunfish (*Lepomis humilis*), redspotted sunfish (*Lepomis miurus*), and bantam sunfish (*Lepomis symmetricus*).

*First drainage records for fish*. First records of six species collected from Patoka River National Wildlife Refuge was previously unknown from the Patoka River (Table 1). These species included skipjack herring, bigeye shiner (*Notropis boops*), silver carp (*Hypophthalmichthys molitrix*), bighead carp, white catfish, and freckled madtom. Skipjack herring is a large river species that is common in the Wabash River. The species was collected from the Patoka River near Meridian Road. The skipjack herring is a pelagic species that is capable of feeding as an adult predator. Bigeye chub was collected from a single site on the Patoka River (at SR 164/162 bridge). This species has experienced significant decline over its range in Illinois and Ohio, but has maintained large populations in Indiana portions of its range. The species is a benthic insectivore that is usually associated with expansive sand bars and coarse gravel and sand substrates. Freckled madtom was collected from Hunley Creek at US 231 bridge. The freckled madtom is a nocturnal species that spends most of its time hiding beneath instream habitat cover. It is possible that this species may have been misidentified in the past since it is similar to several other *Noturus* species that were previously found in the watershed.

*Alien fish species presence*. Silver carp and bighead carp are exotic species from southeast Asia, while white catfish is a non-indigenous species from the Atlantic Slope. These species were all collected from the Patoka River at Oatsville Bottom, while the Asian carps were also collected from the Patoka River upstream of the dam at Winslow. These records represent the first records for these species in the Patoka River. The white catfish was stocked into Patoka Lake and into several other large reservoirs in Indiana near Indianapolis (Simon 2011). The species has a forked tail similar to other members of genus *Ictalurus*, but has white chin barbels and a head shape like other *Ameiurus*. White catfish was only collected from the Patoka River at Oatsville Bottom (Table 1). These species were collected from large main stem river habitats over degraded substrates.

**Big Oaks NWR**

*Fish species richness and composition*. Surveys of streams, lakes, and ponds on the Big Oaks National Wildlife Refuge collected 9,747 individuals representing 37 fish species (Table 1). Dominant families include the Cyprinidae (12 species), Centrarchidae (8 species) and Percidae (5 species). Pruitt et al. (1994) reported collecting 6,703 individuals and dominant families included Cyprinidae (12 species), Centrarchidae (7 species), and Percidae (7 species) during 1993. Historically, bluntnose minnow (1,512 individuals), striped shiner (1,146 individuals), and creek chub (778 individuals) were the three dominant species on the Big Oaks National Wildlife Refuge (Pruitt et al. 1994). During 2006, the dominant species include bluegill (1,159 individuals), bluntnose minnow (964 individuals), creek chub (523 individuals) and central stoneroller (426 individuals). These species were dominant in lakes (bluegill), headwater streams (bluntnose minnow and creek chub), and in large wadable streams (central stoneroller) draining the refuge, respectively (Table 1). During 2007, the dominant species included bluntnose minnow (857 individuals), central stoneroller (751 individuals), creek chub (485 individuals), and striped shiner (461 individuals). These four minnow species were dominant at Little Otter Creek (site 5), Rush Creek, and a variety of sites on Graham Creek (Big and Little Graham Creeks).

*Macroinvertebrate species richness and composition*. A subterranean faunal study was completed by Lewis and Rafail (2002). Lewis and Rafail (2002) documented the cave and spring invertebrate faunas of Big Oaks National Wildlife Refuge. No other surface water surveys of macroinvertebrate assemblages have been completed near the National Wildlife Refuge or in the Vernon Fork of the Muscatatuck River watershed in the vicinity of the refuge. The current study is the first comprehensive evaluation of the Big Oaks National Wildlife Refuge that included taxonomic identification to lowest possible levels. Several cave and spring invertebrate taxa were collected during the surface water surveys. During this investigation of the Big Oaks National Wildlife Refuge watersheds, 163 taxa representing 66 families were collected (Table 3). Dominant families included the Hemiptera, Diptera, and Odonata (8 families), and Coleoptera and Ephemeroptera (7 families). Among the most diverse taxa was the Diptera or flies and midges (35 taxa), Hemiptera or true bugs (25 taxa), Odonata or dragonflies (24 taxa), and Ephemeroptera or mayflies (18 taxa).

Comparison of macroinvertebrate sampling between 2006 and 2007 showed an increase in the number of sensitive taxa were found at the Big Oaks National Wildlife Refuge (Table 3). During the 2006 sampling, 12 unique mayfly taxa including *Baetis flavistriga*, *B. intercalaris*, *Ephemera* spp., *Leucrocuta* spp., *Maccaffertium pulchellum*, *Stenacron* spp., *Isonychia* spp., and *Choroterpes basalis*. During 2007, unique mayfly taxa collected included *Ephemera simulans*, *Attenella attenuate*, *Stenacron interpunctatum*, *Paraleptophlebia* spp. Three stonefly taxa were collected including *Acroneuria* spp., and *Leuctra* spp. during 2006 and *Acroneuria evoluta* during 2007. Six caddisfly species were collected during surveys at Big Oaks National Wildlife Refuge during 2006-2007 (Table 3). Four taxa were unique with two collected in 2006 (*Hydropsyche betteni* and *Chimarra* spp.) and two in 2007 (*Cerclea flava* and *Cernotina spicata*).

*Crayfish species richness and composition*. Limited information about crayfish species is available from the Big Oaks National Wildlife Refuge. Lewis and Rafail (2002) documented the presence of the karst crayfish (*Cambarus laevis*) from the springs and caves occurring on the refuge. St. John (1988) evaluated the distribution of Sloan’s crayfish from areas around the refuge and repeated sampling of select sites in southwestern Ohio (St. John 1991). The results of St. John’s survey resulted in Sloan’s crayfish being considered vulnerable. Thirty four sites were sampled at the Big Oaks National Wildlife Refuge (Suppl. material 1b). These sites represented a wide range of stream sizes from headwater creeks to moderate sized rivers, ponds, and impounded lakes. Seven crayfish species were collected from the Big Oaks National Wildlife Refuge (Table 4). Three primary burrowing species were collected including the paintedhand mudbug, Ortmann’s mudbug, and the Great Plains mudbug. Four species of tertiary burrowing crayfish were collected including Sloan’s crayfish, northern crayfish, Mud River crayfish (*Orconectes juvenilis*), and calico crayfish. The Mud River crayfish is a native species and is known from the study area (Simon 2001). The species resembles the rusty crayfish, but differs in the shape of the mandibles and the first form male gonopod (Taylor 2000). The rusty crayfish is native to the Whitewater River drainage, which is just to the east of the National Wildlife Refuge. No specimens of the rusty crayfish were observed on the refuge; however, specimens were collected from streams that pass through the refuge in areas upstream.

Two orconectid species were collected from only a few sites on the refuge (Table 4). The northern crayfish was collected from two sites on Otter Creek, while the calico crayfish was collected from Little Otter Creek. Both species appear superficially similar; however, the calico crayfish has a deeply incised (notched) dactyl while the northern crayfish does not. There are also differences in the shape and curvature of the first form male gonopod. The northern crayfish reaches much larger sizes and is known to inhabit firm substrates including gravel and cobble substrates, while the calico crayfish inhabits sand and other fine substrates. No crayfish were collected from lentic habitats on the refuge. No crayfish were collected from Old Timbers Lake (site 6), Gate 8 pond (site 14), or Kruegers Lake (site 34). In addition, no crayfish were collected from Big Creek (site 28). The Big Creek site was impounded by a beaver dam and was more lentic than lotic during the time period when sampled. Two attempts to collect crayfish from this site both resulted in no crayfish being collected.

**Changes in Biological Diversity**

*Fish assemblage record changes*. Eleven fish species were collected from the refuge during historical events that were not collected during the current surveys (Pruitt et al. 1994). Longnose gar, bowfin (*Amia calva*), gizzard shad (*Dorosoma cepedianum*), carp (*Cyprinus carpio*), mimic shiner (*Notropis volucellus*), suckermouth minnow (*Phenacobius mirabilis*), spotted sucker, channel catfish (*Ictalurus punctatus*), spotted bass (*Micropterus punctulatus*), logperch (*Percina caprodes*), and blackside darter (*Percina maculata*) were only found during the 1993 surveys. Longnose gar, bowfin, and gizzard shad were collected as single individuals and were found at Blue Hole on Otter Creek. This site was not surveyed during the current investigation due to access restrictions. Carp, an exotic species, was not collected during 2006-2007 surveys, but was found during 1993 as a single individual at Otter Creek and at Graham Creek. Suckermouth minnow, mimic shiner, logperch, and blackside darter were collected from Otter Creek from locations not sampled during the 2006-2007 surveys. These species were either represented by single individuals or were collected from single locations.

During the 2006 sampling, nine species were collected from Big Oaks National Wildlife Refuge that had not been collected during 2007 (Table 5). These species included golden shiner (*Notemigonus crysoleucas*), popeye shiner (*Notropis ariommus*), grass pickerel (*Esox americanus*), brown bullhead (*Ameiurus nebulosus*), blackstripe topminnow, brook silverside, redear sunfish (*Lepomis microlophus*), smallmouth bass, and black crappie. The habitats that these nine fish species were collected include a variety of specific microhabitats. Golden shiner was collected from Big Creek from pool habitat over sand and gravel substrates. This area was associated with a beaver dam that created lentic conditions on Big Creek. Grass pickerel was collected from Little Graham Creek, Marble Creek, an unnamed tributary of Big Creek, and Middle Fork Creek. This species is a pelagic predator that usually is associated with submerged aquatic vegetation, woody debris, and leaf debris. Brown bullhead was collected from Otter Creek from a deep pool along an outside channel bend. The species was associated with large collapsed clay bank habitat that had recently been severed from the bank. Blackstripe topminnow is a surface dwelling species that is commonly associated with overhanging grasses or submerged aquatic vegetation. The species was only collected from Big Creek. Brook silverside is also a pelagic species that usually occurs in lakes; however, the species was collected from Little Graham Creek. Redear sunfish is typically a lake inhabitant that is not native to southeastern Indiana. It has been stocked throughout the state into lentic systems. The species was collected from Old Timbers Lake, Gate 8 pond, and Kruegers Lake, as well as, Little Otter Creek. The species grows to large sizes and is a desirable sport fish among anglers. Likewise, black crappie is also a lake species occurring around woody debris and submerged tree trunks. The species was also collected from Old Timbers Lake, Gate 8 pond, and Kruegers Lake. Smallmouth bass was collected from Otter Creek. This species is a native predator that is an important indicator of water quality because of the temperature sensitivity to cool water temperatures. Twenty-seven of the 36 fish species collected during 2006 were also collected during 2007 (Table 5). The only species that was unique to the 2007 surveys included silver shiner, which was collected from Graham Creek. The silver shiner is a large insectivorous minnow species that is an indicator of high quality habitat and water conditions.

*Alien species presence*. During the 2006-2007 surveys, the western mosquitofish and redear sunfish were collected. The western mosquitofish was collected from three sites on Little Graham Creek (Table 1). This species is widely stocked into ponds and slow moving waters for mosquito control; however, diet studies in Indiana streams has shown that the species consumes snails and other aquatic insects and not mosquitoes (Clem and Whitaker 1996).

**Muscatatuck NWR**

*Fish species richness and composition*. Fifty one species of fish representing 14 families were collected from the 15 sample sites (Table 1). Overall, minnows (Cyprinidae), suckers (Catostomidae), sunfish (Centarchidae), and darters (Percidae) were the most dominate families. Fish assemblage structure differed according to stream size and hydrologic characteristics of each environment. Four lakes were sampled including a moist soil unit (MSU) on refuge property (Suppl. material 1c). All four sites are artificial impoundments and three (Lake Linda, Stansfield Lake, and MSU) have been stocked for sport fishing. Sixteen species belonging to eight families were collected from these sites. The most numerically dominate group at Lake Linda, Stansfield Lake, and MSU was Centarchidae. Bluegill, redear sunfish, and largemouth bass (*Micropterus salmoides*) constituted over three-fourths of the catch with 42.3, 32.9, and 13.6 % of catch, respectively. Largemouth bass (56.1%), bowfin (19.7%), and bluegill (13.2%) were the most dominate fish by weight. These three waterbodies remain level year round and are mostly dominated by stocked fish. The water level in Moss Lake is managed according to season and its fish assemblage differed from the other lentic sites. Moss Lake was sampled during low flow conditions and was heavily vegetated with aquatic macrophytes. In Moss Lake, western mosquitofish, golden shiner, and bowfin were the most numerically dominate fish representing 32.1, 30.4, and 14.8% of catch, respectively. Bowfin also constituted 91% of the catch by relative biomass followed by largemouth bass (4.1%) and bluegill (2%).

Four medium-large wadable streams (>8 m wetted width) were sampled. Mutton Creek was sampled upstream of US 31 bridge where it is a channelized and slow flowing stream. Mutton Creek was dominated by centarchid and catostomid species. Longear sunfish (31.7%), spotted sucker (26.2%), and warmouth (*Lepomis gulosus*) (12.4%) were the most numerically dominant species. Spotted sucker (60.7%), white sucker (11.4%), and bowfin (7.28%) were the most common fish by relative biomass at Mutton Creek. The Vernon Fork Muscatatuck River was sampled at three locations including, upstream and downstream of the refuge and a single site on the refuge. These three sites had similar diverse fish assemblages (Table 1). Thirty nine species from nine families were collected from the Vernon Fork. The most dominant species by number were longear sunfish (27.9%), bullhead minnow (26.4%), and spotfin shiner (*Cyprinella spiloptera*) (6%). The most dominant species at the three Vernon Fork sites were longear sunfish (23.7%), golden redhorse (*Moxostoma erythrurum*) (18.4%), black redhorse (14.5%), silver redhorse (10%), and northern hogsucker (*Hypentelium nigricans*) (9.8%).

Seven small streams (<8 m wetted width) were sampled on refuge property (Suppl. material 1c). Many of these streams were channelized or effected by impoundments and were dominated mostly by cyprinid and centarchid species. Twenty three species were collected at these stream sites (Table 1). Creek chub (50%), central mudminnow (9.8%), green sunfish (*Lepomis cyanellus*) (8.6%), and bluegill (6%) were the most numerically dominate species. Creek chub was also the most dominant species by mass (21.5%) followed by grass pickerel (16.5%), and green sunfish (16%).

*Macroinvertebrate species richness and composition*. The current study is the first comprehensive evaluation of the Muscatatuck National Wildlife Refuge that included taxonomic identifications to lowest possible levels. Eleven sites were sampled for macroinvertebrates (Fig. 3), which correspond to the same sites as sampled for fish assemblages (Suppl. material 1d).

During 2007, nearly 2,505 individuals representing 96 taxa and 45 families were collected from the refuge (Table 3). Dominant families included the Ephemeroptera (6 families), Odonata and Coleoptera (5 families), and Diptera (4 families). Among the most diverse taxa was the Diptera or flies and midges (26 taxa), Coleoptera (10 taxa), and Ephemeroptera and Odonata (9 taxa). The three most dominant taxa in the refuge included isopods and amphipods (Table 3). The dominant taxa included *Lirceus fontinalis* (24%), *Synurella dentata* (16%), and *Hyalella azteca* (14%) (Lewis and Rafail 2002). *Hyalella azteca* is an epibenthic detritivore that occurs in a wide range of habitats. The Ephemeroptera, Plecoptera, and Trichoptera (EPT) taxa are considered among the most sensitive groups of aquatic macroinvertebrates in North American streams (Merritt et al. 2008). Nine mayfly taxa were collected from the Muscatatuck National Wildlife Refuge (Table 3). Taxa sensitive to water quality degradation included *Acerpenna macdunnoughi* and *Eurylophella*. Intermediate mayfly taxa sensitive to degradation includes *Plauditus* and *Leptophlebia*. *Caenis*, *Callibaetis*, *Stenacron*, *Siphlonurus*, and *Stenonema femoratum* are considered tolerant members of the mayfly group (Barbour et al. 1999). These species are capable of tolerating warm water and lower dissolved oxygen levels. Two stonefly taxa include the very sensitive *Isoperla* and *Amphinemura*. Six members of the order Trichoptera were collected including the sensitive *Pycnopsyche* and *Rhyacophila*; and intermediate tolerant *Cheumatopsyche*, *Hydropsyche*, *Ironoquia*, *Ptilostomis* (Barbour et al. 1999).

*Crayfish species richness and composition*. Limited crayfish species information is available from the vicinity of the Muscatatuck National Wildlife Refuge. St. John (1988) and St. John (1991) evaluated the distribution of Sloan’s crayfish from areas around the refuge and repeat sampled select sites in southwestern Ohio. Nineteen sites on the Muscatatuck National Wildlife Refuge were surveyed for crayfish species during the 2007 inventory (Table 4). These sites represented a wide range of stream sizes from headwater creeks to moderate sized rivers, ponds, and impounded lakes.

Six crayfish species were collected from the Muscatatuck National Wildlife Refuge (Table 4). Two primary burrowing species were collected including the paintedhand mudbug and the Great Plains mudbug. The Great Plains mudbug was most common on the refuge occurring at 14 sites (73.7% sites). A blue form of the Great Plains mudbug was collected downstream of the Stanfield Lake outlet (site 16). The habitat had a large number of burrows and the soil was grey in color. The blue-form crayfish when left in the sun returned to the typical olive green and brown coloration suggesting perhaps a vitamin deficiency. Two secondary burrowing species were collected from the refuge. The karst crayfish is typical of springs and cave streams. The species was collected from the unnamed tributary of Storm Creek (site 19), while the White River crayfish was collected from Sandy Branch (site 13). Two tertiary burrowing crayfish species were collected including Sloan’s crayfish and the calico crayfish.

*Alien species presence*. The only non-indigenous species collected on the Muscatatuck National Wildlife Refuge was the western mosquitofish. It was collected from Linda (site 1) and Moss lakes (site 2), Mutton Creek, Sandy Branch, and the Vernon Fork Muscatatuck River downstream of the refuge (Table 1). The western mosquitofish is intentionally stocked for mosquito control. The species is not effective for controlling mosquitoes in flowing waters and may be only marginally successful in lakes and ponds (Clem and Whitaker 1996).

## Discussion

Maintaining or restoring biological integrity is not the same as maximizing biological diversity (Angermeier and Karr 1994). Maintaining biodiversity may entail managing for a single species or community at some refuges and combinations of species or communities at other refuges. This paradigm shift will provide a role for resistance and resilience management (Carpenter and Brock 2004, Glicksman and Cumming 2012). For example, a refuge may contain critical habitats for an endangered species. Maintaining that habitat (and, therefore, that species), even though it may reduce biological integrity at the refuge scale, helps maintain biodiversity at the ecosystem or national landscape scale.

In deciding which management activities needs to be conducted to accomplish refuge purpose(s) while maintaining biological integrity, we consider how the ecosystem functioned under historic conditions (Ehlers 2014). For example, to maintain certain habitats implementation of natural frequency and timing of processes, such as flooding, fires, and grazing would be required. Where it is not appropriate to restore ecosystem function, refuge management will attempt to duplicate these natural processes including natural frequencies and timing to the extent this can be accomplished (Meretsky et al. 2006).

Landscape diversity is descriptive of the number and dominance of different patch types and is a fundamental component of refuge management (Peters and Goslee 2001). It may be necessary to modify the frequency and timing of natural processes at the refuge scale to fulfill refuge purpose(s) or to contribute to biological integrity at larger landscape scales (Ehlers 2014). Many wetlands have been converted to agriculture or other land uses and the remaining wetlands must produce more habitat, more consistently, to support wetland-dependent species. Therefore, to conserve populations at larger landscape scales, we may flood areas more frequently and for longer periods of time than they were flooded historically.

**Changes in Biological Integrity**

Angermeier and Karr (1994) recommend that the focus for watershed management should be towards biological integrity as an overarching ecological organizational hierarchy. Aquatic systems are appropriate models for managing ecological consequences of anthropogenic impacts at the landscape level since rates of biodiversity decline for aquatic fauna exceeds those for terrestrial fauna (Williams and Neves 1992). Refuges are unlikely to sustain all biodiversity or even all species, thus partnerships between government agencies and the public are essential.

**Figure 5.**
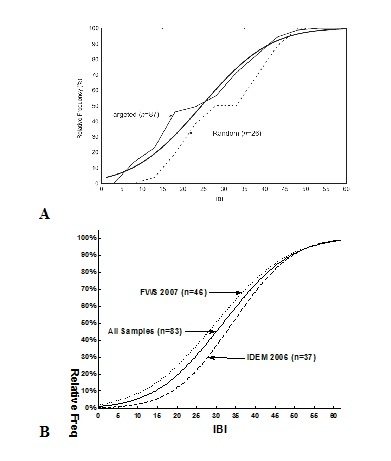
Comparison of Relative IBI Cumulative Distribution Frequency for sites sampled within the Patoka River drainage including: A) Fish & Wildlife Service 2001 and B) Fish & Wildlife Service 2007 (n=46) and the Indiana Department of Environmental Management in 2006 (n=37) and a combination of all sites (n=83) (Suppl. material 2).

*Patoka River NWR*. In order to determine the biological integrity and ecological health of the Patoka River National Wildlife Refuge, we chose an unbiased approach to verify our understanding of overall biological integrity (Simon et al. 1995, Simon et al. 2005a). Biological integrity classification scores, based on targeted least-impacted sampling at 34 sites between 1992 and 2001 (Simon et al. 2005a), showed that stream biological integrity of the Patoka River National Wildlife Refuge and associated watersheds had declined slightly over this period (Fig. 5a) compared to previous sampling events. Simon et al. (2005a) reported that watershed integrity decline reached the lowest levels recorded since 1888.

The probability distributions of biological integrity, based on index of biotic integrity (IBI) score for the watershed showed that the two years had similar results (Fig. 5b). The results from 2006 were slightly higher than scores from 2007, but this is to be expected because of the drought conditions that occurred in 2007. The two years showed that site mean cumulative frequency distribution (CFD_50_) had higher biological integrity during 2006 with IBI scores of 35, which approximates the statewide average for Indiana, while the CFD_50 _for 2007 had mean biological integrity scores of 31. Both integrity categories would have scored between “Poor-Fair” based on index classification assessments (Karr 1981, Simon and Dufour 1998). Based on the assessment of all 83 sample events collected at Patoka River, the mean CFD_50 _scored 32 using the IBI (Simon et al. 2005a).

The trend for biological integrity in the Patoka River National Wildlife Refuge is not significantly different from previous surveys conducted between 1993—2001 (Simon et al. 1995, Simon et al. 2005a). IBI scores from 1993 surveys averaged 21 (range: 0-48) and represented “very poor” IBI biological integrity class. During 2001, the mean IBI score was 17 (range: 0-42). The trend in IBI score has had a positive slope and has slightly improved since the original watershed survey in 1993. Survey results based on 2006 and 2007 sampling showed that the watershed has improved enough to meet the statewide average. A variety of sites do not possess any fish species in the South Fork Patoka River, which is an area that is impacted from acid mine drainage, as well as Rough Creek, Pike County, which was also without aquatic life (Simon et al. 1995). Based on study periods from 2001 to 2006-2007, conditions favored a slight increase in biological integrity of streams in the Patoka River watershed (Simon et al. 2005a). Over this time period, drought conditions possibly reduced nonpoint source runoff of nutrients and toxic materials into streams, while groundwater infiltration has potentially enabled some species to recolonize areas that had been in past decline. Unfortunately, the lack of water in 2007 caused some loss of biological integrity gain with declines in species richness and changes in trophic dynamics.

**Figure 6.**
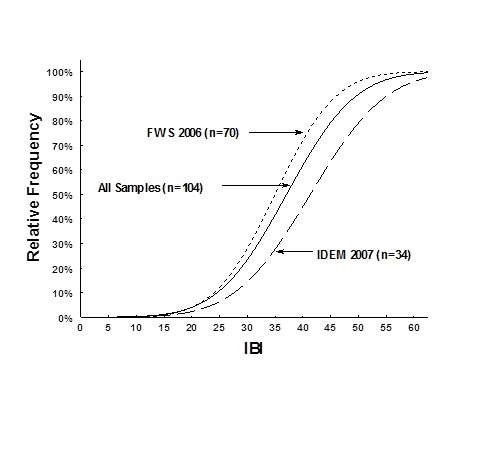
Comparison of Relative IBI Frequency for sites sampled by Fish & Wildlife Service in 2006 (n=70), the Indiana Department of Environmental Management in 2007 (n=34), and a combination of all sites (n=104) within and around the Big Oaks National Wildlife Refuge (Suppl. material 3).

*Big Oaks NWR*. Biological integrity classification scores, based on targeted least-impacted sampling at 34 sites between 1992 and 2001, showed that stream biological integrity of the Big Oaks National Wildlife Refuge and associated watersheds had declined slightly over this period (Fig. 6). Over this time period, prolonged drought conditions possibly reduced nonpoint source runoff of nutrients and toxic materials into streams, while groundwater infiltration has potentially enabled some species to recolonize areas that had been decimated in the past (U.S. Geological Survey Water Resources data, 2004-2006). Unfortunately, the lack of water also caused declines in species richness and changes in trophic dynamics.

At the Big Oaks National Wildlife Refuge we sampled 14 sites. We did not sample lake or pond locations in 2007 that had been prior sampled during 2006. The probability distributions of biological integrity, based on index of biotic integrity (IBI) score for the watershed showed that the two years had similar results (Fig. 6). The results from 2007 had several sites that had higher integrity scores than the 2006 random sites, but this is to be expected because of the drought situation. The two years showed that site mean cumulative frequency distribution (CFD_50_) had higher biological integrity with IBI scores of 41, while the CFD_50 _for 2006 had biological integrity scores of 35. Both integrity categories would have scored between “Poor-Fair” based on index classification assessments (Karr 1981, Simon and Dufour 1998). Based on the assessment of all 104 sample events collected at Big Oaks, the mean CFD_50 _would have scored 37 using the IBI (Simon and Dufour 1998).

The trend for biological integrity in the Big Oaks National Wildlife Refuge is not significantly different during the surveys conducted between 1993 to 2007 (Pruitt et al. 1994, Simon 2008). IBI scores from 1993 sampling averaged 46 (range: 32-58) and represented “good-fair” integrity classes of biological integrity. Although the trend has slightly declined since the historical surveys, this is most likely a result of including the lake sites in the IBI statistics and the emphasis on higher order streams in the 1993 surveys. Larger streams such as Otter Creek represented 23.5% of the 1993 collections compared to 5.9% of the 2006 and none of the 2007 collections. Since the 1993 stream sites were not randomly selected, there was a greater opportunity to target highest quality habitats. The dynamic change in biodiversity over time requires management actions promoting native species structure and function (Noss 2004).

*Muscatatuck NWR.* The seven small, wadable streams sampled on the refuge ranged from "poor" to "fair" (Suppl. material 1c) when compared to reference conditions for the Eastern Corn Belt Plain ecoregion. Index of Biotic Integrity scores ranged from 26 to 34 for these stream sites. The low IBI scores are largely a result of hydrologic modifications to the aquatic habitat on refuge to benefit migratory waterfowl and sport fishery. These streams are dominated by sunfish and bass species and lack sensitive sucker and darter species resulting from habitat modification and stocking of lakes for sport fishing.

The larger streams showed higher quality biological conditions. The four larger stream sites ranged from "very good" to "exceptional" (Suppl. material 1c). Scores ranged from 46 to 56 with two of the Vernon Fork sites scoring "exceptional." These sites supported populations of sensitive minnow species, such as bigeye chub, sucker species including golden redhorse, black redhorse, northern hogsucker; and several sensitive darter species including greenside (*Etheostoma blennioides*), rainbow darter (*E. caeruleum*), harlequin darter, logperch, dusky darter, and eastern sand darter. Hydrologic modifications on refuge have had little impact on the Vernon Fork and the river continues to support a high quality assemblage of native species.

The four lakes ranged from “fair” to “fair-good” (Suppl. material 1). Both Lake Linda and Moss Lake were considered “fair”, while the MSU and Lake Stansfield were both considered “fair-good”. The lack of benthic species was the primary reason for the lower sustainability score in Lake Linda and Moss Lake.

**Figure 7.**
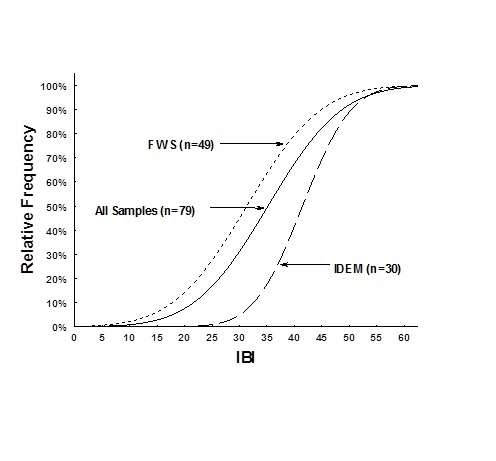
Comparison of Relative IBI Cumulative Distribution Frequency for sites sampled by Fish & Wildlife Service (n=49), the Indiana Department of Environmental Management (n=30), and a combination of all sites (n=79) within and around the Muscatatuck National Wildlife Refuge during 2007 (Suppl. material 4).

The cumulative frequency distributions did not include the lake or pond sites that had been sampled during 2006. Based on probability distributions of biological integrity, index of biotic integrity (IBI) score for the watershed (Fig. 7) showed that the two sampling periods had a wide range of results. The earlier sampling (N=49) showed lower integrity than sites sampled later in the summer (N=30). This was expected as the drought conditions concentrated fish into isolated pools. The two sampling periods showed that site mean reach cumulative frequency distribution (CFD_50_) in the early summer (June) had lower biological integrity with IBI scores of 31, while the CFD_50 _for late summer (August) had mean biological integrity reach scores of 40, which was above the statewide average for Indiana. The early summer integrity category was considered “Poor”, while the later summer integrity class would have been considered “fair-good” based on index classification assessments (Karr 1981, Simon and Dufour 1998). Based on the assessment of all 79 sample events collected at Big Oaks, the mean CFD_50 _would have scored 35 using the IBI (Simon and Dufour 1998).

Although the trend has slightly improved between the two surveys periods in 2007, this is most likely a result of not including the lake sites in the IBI statistics and the greater number of higher order streams in the later summer sampling events. Larger streams such as Vernon Fork represented a higher percentage of the collections compared to 4.08% of the early summer collections. A different random draw was selected between the two periods so that only a few of the samples included the same sites. The survey results based on the 2007 survey is probably most representative of the variety of aquatic habitat conditions found at Muscatatuck National Wildlife Refuge.

**Figure 8.**
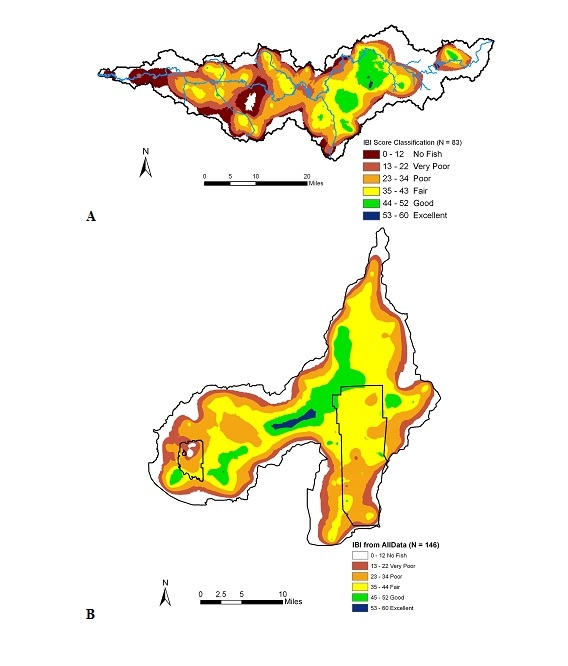
Watershed scale biological integrity spline smoothed pleths for the Patoka River (A) and Muscatatuck River (B) watershed based on reach level Index of Biotic Integrity (IBI) scores (Suppl. materials 2, 3, 4).

Landscape level management for wholeness, resistence, and resilience requires the recognition of network patterns within the basin management planning (Noss 2004). Watershed patterns in biological integrity for the two drainage areas showed that the Muscatatuck River drainage had higher biological integrity than the Patoka River drainage (Fig. 8). The Patoka River had the greatest variation in IBI scores with ranges between 0-56 (Table 6). The highest biological integrity was associated with Hoosier National Forest and the tributaries draining into Patoka Lake (Fig. 8a); however, the highest reach scale IBI score was a score of 56 IBI points that was associated with tributaries of the Hoosier National Forest. The highest percentage of no fish and very poor IBI conditions were found in the lower Patoka River and were associated with Sugar Ridge State Recreation Area and the Patoka River NWR. The lower Patoka River also exhibited a high proportion of degraded conditions.

Biological integrity associated with the Muscatuck River drainage was highest in the area where the Vernon Fork joined the mainstem Muscatatuck River (Fig. 8b). Most of the remainder of the watershed was considered fair condition. Only a few areas within the Muscatatuck River drainage had no fish, both were associated with a treatment plant pump overflow that drained raw sewerage into the refuge along Storm and Mutton Creeks.

**Species richness and watershed patterns**

The aquatic fauna of Indiana’s National Wildlife Refuges includes a significant portion of the rarest of Indiana’s fish fauna; however, due to contaminant impacts associated with legacy land use the refuges are not necessarily considered least-impacted habitats (Simon and Morris 2009). An analysis of the highest species richness areas within the refuges show that most of the biodiversity is attributed to macroinvertebrate taxa richness (Table 6). The Patoka River NWR had the highest species richness of both macroinvertebrates and fish, while Big Oaks NWR had the highest species richness of crayfish species.

**Figure 9.**
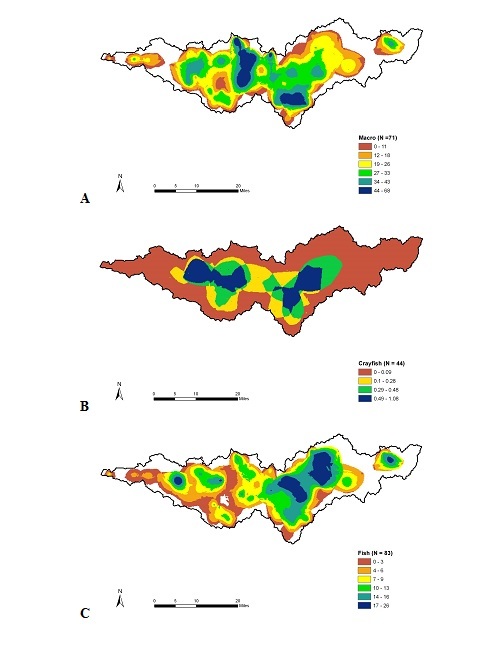
Watershed scale biodiversity pleths for the Patoka River drainage based on spline smoothed joined means of assemblage structure. A. macroinvertebrates based on taxa richness, B. crayfish based on Shannon-Weiner diversity index, and C. fish based on species richness (Suppl. material 2).

**Figure 10.**
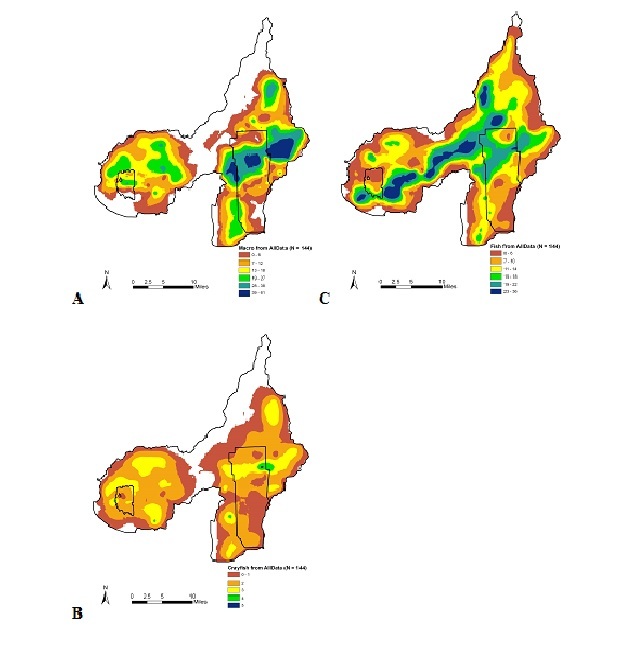
Watershed scale biodiversity spline smoothed pleths for the Muscatatuck River drainage based on species richness joined means of assemblage structure. A. macroinvertebrates, B. crayfish, and C. fish (Suppl. materials 3, 4).

A hot-spot analysis of watershed biodiversity showed that the central portion of the Patoka River drainage had the highest macroinvertebrate species richness (Fig. 9a), which was associated with the Flat Creek, Green Creek, and the Patoka River. The highest crayfish species richness was associated with the areas of the Patoka River that drained Hoosier National Forest and was downstream of Patoka Lake and lower Patoka River and Hurricane Creek (Fig. 9b). The highest species richness for fish assemblages was associated with areas draining Hoosier National Forest and surrounding Patoka Lake (Fig. 9c). A similar hot-spot analysis of watershed biodiversity patterns for the Muscatatuck River drainage showed that the highest species richness of macroinvertebrates (Fig. 10a) and crayfish (Fig. 10b) occurred within Graham Creek of Big Oak NWR. The highest fish species richness was associated with the mainstem channel of the Muscatatuck River and associated tributary mouths (Fig. 10c).

The management goal of sustaining ecological and evolutionary processes within a natural range of variability requires an understanding of the fundamental elements of the landscape (Noss 2004). Surveys of National Wildlife Refuges provide opportunities to determine species composition, species richness, and promote the management of biodiversity, while landscape level planning can evaluate the biological integrity of watershed levels. This incorporation of reach and larger landscape scales promotes effective conservation management of vital aquatic resources.

## Supplementary Material

Supplementary material 1 Supplemental Materials Appendix: Sites Data type: Location georeferenced information
Brief description: Site distribution data with decimal minute degree lat-lon for study sites.
File: oo_34397.docx Simon, T.P.

Supplementary material 2 Comparison of Relative IBI Cumulative Distribution Frequency for sites sampled within the Patoka River drainage Data type: Analyzed IBI score calculations
Brief description: Patoka raw data on separate spreadsheets including macroinvertebrate, crayfish, and fish information and analyzed results of index of biotic integrity, Shannon-Weiner, and species richness.
File: oo_34508.xls TP Simon & CC Morris

Supplementary material 3 Comparison of Relative IBI Frequency for sites sampled by Fish & Wildlife Service in 2006 (n=70), the Indiana Department of Environmental Management in 2007 (n=34), and a combination of all sites (n=104) within and around the Big Oaks National Wildlife Refuge Data type: Raw and Analyzed data for Big Oaks National Wildlife Refuge
Brief description: Data files for fish, crayfish, macroinvertebrate, QHEI, index of biotic integrity, Shannon-Weiner, and Species richness information for the Big Oaks National Wildlife Refuge.
File: oo_34509.xls TP Simon & CC Morris

Supplementary material 4 Comparison of Relative IBI Cumulative Distribution Frequency for sites sampled by Fish & Wildlife Service (n=49), the Indiana Department of Environmental Management (n=30), and a combination of all sites (n=79) within and around the Muscatatuck National Wildlife Refuge during 2007. Data type: Raw and Analyzed data for Muscatatcuck National Wildlife Refuge
Brief description: Data files for fish, crayfish, macroinvertebrates, including analyzed information for QHEI, index of biotic integrity, Shannon-Weiner, and Species richness for the Muscatatuck National Wildlife Refuge
File: oo_34523.xls TP Simon & CC Morris

XML Treatment for Synurella dentata

XML Treatment for Lirceus fontinalis

XML Treatment for Orconectes (Faxonius) indianensis

XML Treatment for Orconectes (Rhoadesius) sloanii

XML Treatment for Notropis ariommus

XML Treatment for Centrarchus macropterus

XML Treatment for Lepomis (Lepomis) symmetricus

XML Treatment for Ammocrypta (Ammocrypta) pellucida

XML Treatment for Etheostoma (Etheostoma) histrio

## Figures and Tables

**Table 1.** List of fish species collected from three National Wildlife Refuges in southern Indiana. Numbers indicate the sites at which each species has been collected based on information in Supplemental materials Suppl. material 1 (Appendix a, b, and c) and Figs 1, 2, 3.

Species	Patoka River	Muscatatuck	Big Oaks
Petromyzontidae			
*Lethenteron appendix* (DeKay 1842)		6, 11	
Lepisosteidae			
*Lepisosteus oculatus* Winchell 1864	6		
*Lepisosteus osseus* (Linnaeus 1758)	10	14	
*Lepisosteus platostomus* Rafinesque 1820	20, 34, 61, 74, 76, 78		
Amiidae			
*Amia calva* Linnaeus 1766	6, 20, 33, 34, 36, 55, 68, 69	2, 3, 5	
Hiodontidae			
*Hiodon alosoides* (Rafinesque 1819)	61		
Clupeidae			
*Alosa chrysochloris* (Rafinesque 1820)	63		
*Dorosoma cepedianum* (Lesueur 1818)	1, 2, 6, 7, 9, 11-13, 16, 20, 33, 34, 61, 63, 74, 78		
*Dorosoma petenense* (Günther 1867)	61		
Cyprinidae			
*Campostoma anomalum* (Rafinesque 1820)	1-5, 8, 9, 13-22, 24-27, 29-30, 35, 38, 45, 47, 48, 50-51, 64-65, 72-73	6, 8, 14	1-5, 7-13, 15-16, 20-27, 29-32
*Chrosomus erythrogaster* (Rafinesque 1820)	4		
*Cyprinella spiloptera* (Cope 1867)	2, 6, 7, 9-13, 16, 19, 20, 25-27, 32, 34, 42, 74, 78	6, 14-15	5, 12, 20
*Cyprinella whipplei* Girard 1856	9, 12, 16, 19, 20, 44, 63,	6, 14-15	
*Cyprinus carpio* Linnaeus 1758	2, 6, 7, 12, 33, 34, 36, 43, 63, 67, 74, 76-78		
*Ericymba buccata* Cope 1865	12-14, 16, 18-21, 25-27, 29, 30, 35, 44-45, 47-48, 50, 73	13, 15	1, 2, 5, 7-13, 26
*Hybognathus nuchalis* Agassiz 1855	20, 63, 65, 66		
*Hybopsis amblops* (Rafinesque 1820)	12	15	1-3, 5, 8-12
*Hypophthalmichthys molitrix* (Valenciennes 1844)	36, 61, 74		
*Hypophthalmichthys nobilis* (Richardson 1845)	74		
*Luxilus chrysocephalus* (Rafinesque 1820)	1-3, 7-9, 12-14, 18-19, 21, 22, 45, 61, 66		1, 5, 7-13, 15, 20-21, 24-27, 29-30, 32
*Lythrurus fumeus* (Evermann 1892)	6, 7, 10, 42, 51		
*Lythrurus umbratilis* (Girard 1856)	1-3, 5, 6, 8, 9, 13, 19, 20, 25-27, 31, 34, 42, 61	6, 11, 14, 15	2, 8-12, 17, 19-22, 24, 30
*Notemigonus crysoleucas* (Mitchill 1814)	28, 31, 40, 45	1, 2, 4, 10, 12	28
*Notropis ariommus* (Cope 1867)			1, 11, 20
*Notropis atherinoides* Rafinesque 1818	54, 63, 74, 77	15	
*Notropis boops* Gilbert 1884			1, 2, 8-10, 12, 20, 29-30
*Notropis photogenis* (Cope 1865)			8, 10
*Phenacobius mirabilis* (Girard 1856)	12-13, 16, 26-27, 34, 72-74		
*Pimephales notatus* (Rafinesque 1820)	1-9, 12-14, 16, 18-23, 25-32, 34, 38, 42, 45, 47, 61, 63, 66, 74, 75	6, 8, 11, 13-15	1-5, 7-13, 16, 20-24, 26, 28-33
*Pimephales promelas* Rafinesque 1820	4, 8, 75		
*Pimephales vigilax* (Baird and Girard 1853)	12, 61	6, 14-15	
*Semotilus atromaculatus* (Mitchill 1818)	1, 3-5, 8, 9, 13-15, 17-19, 21-22, 24-25, 27-31, 35, 39, 40, 42, 44-51, 54, 56-58, 60, 64-66, 70, 71, 73-74	8-11, 14-15	2-5, 7-13, 15-33
Catostomidae			
*Carpiodes carpio* (Rafinesque 1820)	10, 33, 74		
*Carpiodes cyprinus* (Lesueur 1817)	45, 61		
*Catostomus commersonii* (Lacepède 1803)	1-4, 8, 13, 17, 19	5, 8, 11	3, 5, 8-13, 28, 32-33
*Erimyzon oblongus* (Mitchill 1814)	3, 8, 13-14, 19, 20, 22-23, 26, 35, 40, 42		7, 18-21, 24-27, 29
*Erimyzon sucetta* (Lacepède 1803)	18, 21, 25, 27-32, 45		
*Hypentelium nigricans* (Lesueur 1817)		5, 14-15	1, 2, 8-12, 23, 30
*Ictiobus bubalus* (Rafinesque 1818)	6, 10, 11, 33, 61, 63, 74, 76		
*Ictiobus cyprinellus* (Valenciennes 1844)	6, 10, 33, 74		
*Ictiobus niger* (Rafinesque 1819)	6, 7, 10, 11, 33, 34, 36, 63, 78		
*Minytrema melanops* (Rafinesque 1820)	2, 6, 9, 13, 16, 19	2, 3, 5, 15	
*Moxostoma anisurum* (Rafinesque 1820)		5, 6, 15	
*Moxostoma duquesnei* (Lesueur 1817)	11	3, 5, 6, 14-15	1, 2, 8-12, 20
*Moxostoma erythrurum* (Rafinesque 1818)	1, 2, 6, 7, 9, 12, 16, 19	5, 14-15	1, 2, 5, 8, 10, 11
*Moxostoma macrolepidotum* (Lesueur 1817)	61		
Ictaluridae			
*Ameiurus catus* (Linnaeus 1758)	74		
*Ameiurus melas* (Rafinesque 1820)	38, 62, 68, 69, 71	9	
*Ameiurus natalis* (Lesueur 1819)	4, 5, 8, 9, 13-14, 16, 18-22, 26-28, 30, 32, 38-40, 42, 44-48, 50-52, 54, 57, 62, 64-66, 72, 75	5, 7, 9-10, 12	1, 5, 9-13, 15, 28-29, 33
*Ameiurus nebulosus* (Lesueur 1819)	6, 13, 19, 26, 39, 40, 42, 46	3, 15	1
*Ictalurus punctatus* (Rafinesque 1818)	11, 12, 20, 23, 34, 54, 61, 74, 77		
*Noturus flavus* Rafinesque 1818	78		
*Noturus gyrinus* (Mitchill 1817)	25, 45		
*Noturus miurus* Jordan 1877	12	6, 14-15	9-10, 20
*Noturus nocturnus* Jordan and Gilbert 1886	32		
*Pylodictis olivaris* (Rafinesque 1818)	6, 12, 33, 34, 54, 74, 78		
Esocidae			
*Esox americanus vermiculatus* Lesueur 1846	20, 35, 38-40, 51, 54, 55, 59, 64-66, 74	2, 6, 7, 10-13	13, 16, 27, 30
Umbridae			
*Umbra limi* (Kirtland 1841)	65, 66	7, 9, 11, 13	
Aphredoderidae			
*Aphredoderus sayanus gibbosus* Lesueur 1833	2, 7, 8, 11, 20, 23, 26, 31, 32, 34, 38-40, 42, 43, 48, 51, 59, 65	2, 10	
Fundulidae			
*Fundulus notatus* (Rafinesque 1820)	1-3, 5, 9, 12-14, 16, 18-23, 25-31, 38-41, 45-48, 51-56, 58-59, 61, 64-68, 71-73, 75	13	20
Poeciliidae			
*Gambusia affinis affinis* (Baird and Girard 1853)	12, 14, 16, 18, 20-21, 25-28, 30-35, 37, 44-47, 51, 53, 56, 58-59, 64, 66-67, 69-71, 73, 75, 77, 78	1, 2, 12-14	12-13, 15
Atherinidae			
*Labidesthes sicculus* (Cope 1865)	6, 12, 13, 16, 20, 61, 74	12	13
Cottidae			
*Cottus carolinae* (Gill 1861)	1, 3		
Centrarchidae			
*Ambloplites rupestris* (Rafinesque 1817)	1, 2	15	1-2, 11-12
*Centrarchus macropterus* (Lacepède 1801)	62, 68	2, 5, 12	
*Lepomis cyanellus* Rafinesque 1819	1-9, 12-14, 16-22, 25-28, 30-31, 35, 37-40, 42-56, 58-59, 62, 64-75	2, 5-15	1-5, 7, 9-13, 15-22, 24-30, 32-33
*Lepomis gulosus* (Cuvier 1829)	1, 2, 5, 12, 59, 62, 70	1-5, 7, 9-12, 14	
*Lepomis humilis* (Girard 1858)	12		
*Lepomis macrochirus* Rafinesque 1819	1-5, 8-14, 16-17, 19-23, 25-26, 33-40, 42-44, 46-48, 50-52, 54-55, 60-66, 68, 70, 74, 76	1-5, 7, 9-12, 14-15	1, 2, 5, 6, 8-15, 17, 19-20, 28-29, 32, 34
*Lepomis megalotis* (Rafinesque 1820)	1-14, 16, 18-23, 25-28, 32-35, 38-40, 43, 44, 47-48, 50-55, 59-64, 66, 74	5-6, 9, 11, 13-15	1-7, 8-17, 26-27, 29-30
*Lepomis microlophus* (Günther 1859)	1, 9, 20, 59, 62, 65	1-4	5-6, 14, 34
*Lepomis miniatus* (Jordan 1877)	62	2	
*Lepomis symmetricus* Forbes 1883	52		
*Micropterus dolomieu* Lacepède 1802	1, 2, 7		1
*Micropterus punctulatus* (Rafinesque 1819)	5-8, 12, 17, 19, 20, 28, 32, 40, 47, 61, 64, 66, 74, 77	6, 15	
*Micropterus salmoides* (Lacepède 1802)	1, 2, 6, 7, 9, 13, 16, 22-23, 26, 38, 46, 54, 67, 69, 72, 75	1-5, 9-10, 12	2, 6, 11, 14-15, 34
*Pomoxis annularis* Rafinesque 1818	1, 2, 11, 20, 36, 63		
*Pomoxis nigromaculatus* (Lesueur 1829)	33, 36	3-5	6, 14, 34
Percidae			
*Ammocrypta pellucida* (Putnam 1863)		15	
*Etheostoma asprigene* (Forbes 1878)		5-6	
*Etheostoma asprigene* nov. sp	32, 38, 74		
*Etheostoma blennioides* Rafinesque 1819			1-2, 5, 8-12, 20, 29-30
*Etheostoma caeruleum* Storer 1845		14	1-3, 5, 8-13, 20, 25-26, 29, 30
*Etheostoma flabellare* Rafinesque 1819			1-5, 7-13, 16, 20, 23-25, 29, 30
*Etheostoma gracile* (Girard 1859)	31-32, 34, 37, 65		
*Etheostoma histrio* Jordan and Gilbert 1887	78	6, 14	
*Etheostoma nigrum* Rafinesque 1820	1-3, 14, 42, 45	6, 11, 13-15	1-5, 7-13, 15-16, 20-26, 28-30
*Etheostoma spectabile* (Agassiz 1854)	1, 3-5, 8, 9, 14, 18-19, 21-22, 27		2-5, 7-13, 15-17, 21-25, 27, 29, 30, 32-33
*Percina caprodes* (Rafinesque 1818)	7, 12-13	5, 15	
*Percina maculata* (Girard 1859)	7, 19, 39, 42, 48	15	
*Percina phoxocephala* (Nelson 1876)	11, 16	6, 14, 15	
*Percina sciera* (Swain 1883)	7, 10, 11-12, 39, 74	6, 14-15	
Sciaenidae			
*Aplodinotus grunniens* Rafinesque 1819	7, 11-12, 20, 33, 34, 36, 63, 76, 78		

**Table 2.** Comparison of fish assemblage structure and catch percentages from the Patoka River drainage, 2006 to 2007.

	2006		2007		Total	
Species	Count	%	Count	%	Count	%
Lepisosteidae						
*Lepisosteus oculatus*	1	<1%			1	<1%
*Lepisosteus osseus*	1	<1%			1	<1%
*Lepisosteus platostomus*	11	<1%	5	<1%	16	<1%
Amiidae						
*Amia calva*	9	<1%	3	<1%	12	<1%
Hiodontidae						
*Hiodon alosoides*			1	<1%	1	<1%
Clupeidae						
*Alosa chrysochloris*	1	<1%			1	<1%
*Dorosoma cepedianum*	90	2%	72	2%	162	2%
*Dorosoma petenense*			2	<1%	2	<1%
Cyprinidae						
*Campostoma anomalum*	827	16%	323	7%	1150	12%
*Chrosomus erythrogaster*	3	<1%			3	<1%
*Cyprinella spiloptera*	142	3%	89	2%	231	2%
*Cyprinella whipplei*	70	1%	2	<1%	72	1%
*Cyprinus carpio*	47	1%	39	1%	86	1%
*Ericymba buccata*	21	<1%	203	4%	224	2%
*Hybognathus nuchalis*	12	<1%	70	2%	82	1%
*Hybopsis amblops*			1	<1%	1	<1%
*Hypophthalmichthys molitrix*	1	<1%	6	<1%	7	<1%
*Hypophthalmichthys nobilis*			3	<1%	3	<1%
*Luxilus chrysocephalus*	378	7%	71	2%	449	5%
*Lythrurus fumeus*	5	<1%			5	<1%
*Lythrurus umbratilis*	75	1%	10	<1%	85	1%
*Notemigonus crysoleucas*	1	<1%	50	1%	51	1%
*Notropis atherinoides*	6	<1%	8	<1%	14	<1%
*Phenacobius mirabilis*	7	<1%	81	2%	88	1%
*Pimephales notatus*	283	6%	524	12%	807	8%
*Pimephales promelas*	2	<1%	2	<1%	4	<1%
*Pimephales vigilax*			27	1%	27	<1%
*Semotilus atromaculatus*	210	4%	671	15%	881	9%
Catostomidae						
*Carpiodes carpio*	2	<1%	1	<1%	3	<1%
*Carpiodes cyprinus*			2	<1%	2	<1%
*Catostomus commersonii*	89	2%			89	1%
*Erimyzon oblongus*	114	2%	7	<1%	121	1%
*Erimyzon sucetta*			209	5%	209	2%
*Ictiobus bubalus*	18	<1%	8	<1%	26	<1%
*Ictiobus cyprinellus*	4	<1%	4	<1%	8	<1%
*Ictiobus niger*	28	1%			28	<1%
*Minytrema melanops*	21	<1%			21	<1%
*Moxostoma duquesnei*	1	<1%			1	<1%
*Moxostoma erythrurum*	31	1%	1	<1%	32	<1%
*Moxostoma macrolepidotum*			1	<1%	1	<1%
Ictaluridae						
*Ameiurus catus*			1	<1%	1	<1%
*Ameiurus melas*	1	<1%	22	<1%	23	<1%
*Ameiurus natalis*	141	3%	69	2%	210	2%
*Ameiurus nebulosus*	17	<1%	1	<1%	18	<1%
*Ictalurus punctatus*	8	<1%	11	<1%	19	<1%
*Noturus flavus*	4	<1%			4	<1%
*Noturus gyrinus*			2	<1%	2	<1%
*Noturus miurus*			1	<1%	1	<1%
*Noturus nocturnus*			2	<1%	2	<1%
*Pylodictis olivaris*	5	<1%	5	<1%	10	<1%
Esocidae						
*Esox americanus*	17	<1%	13	<1%	30	<1%
Umbridae						
*Umbra limi*			7	<1%	7	<1%
Aphredoderidae						
*Aphredoderus sayanus*	19	<1%	24	1%	43	<1%
Fundulidae						
*Fundulus notatus*	58	1%	423	9%	481	5%
Poeciliidae						
*Gambusia affinis*	33	1%	568	12%	601	6%
Atherinidae						
*Labidesthes sicculus*	15	<1%	5	<1%	20	<1%
Cottidae						
*Cottus carolinae*	24	<1%			24	<1%
Centrarchidae						
*Ambloplites rupestris*	9	<1%			9	<1%
*Centrarchus macropterus*			2	<1%	2	<1%
*Lepomis cyanellus*	260	5%	288	6%	548	6%
*Lepomis gulosus*	5	<1%	5	<1%	10	<1%
*Lepomis humilis*			1	<1%	1	<1%
*Lepomis macrochirus*	461	9%	178	4%	639	7%
*Lepomis megalotis*	1314	26%	264	6%	1578	16%
*Lepomis microlophus*	8	<1%	7	<1%	15	<1%
*Lepomis miniatus*			11	<1%	11	<1%
*Lepomis symmetricus*			1	<1%	1	<1%
*Micropterus dolomieu*	7	<1%			7	<1%
*Micropterus punctulatus*	26	1%	57	1%	83	1%
*Micropterus salmoides*	25	<1%	11	<1%	36	<1%
*Pomoxis annularis*	22	<1%			22	<1%
*Pomoxis nigromaculatus*	2	<1%			2	<1%
Percidae						
*Etheostoma asprigene* nov. sp.	1	<1%	2	<1%	3	<1%
*Etheostoma gracile*	2	<1%	41	1%	43	<1%
*Etheostoma histrio*	1	<1%			1	<1%
*Etheostoma nigrum*	8	<1%	3	<1%	11	<1%
*Etheostoma spectabile*	74	1%	17	<1%	91	1%
*Percina caprodes*	2	<1%	1	<1%	3	<1%
*Percina maculata*	5	<1%	1	<1%	6	<1%
*Percina phoxocephala*	2	<1%			2	<1%
*Percina sciera*	5	<1%	3	<1%	8	<1%
Sciaenidae						
*Aplodinotus grunniens*	18	<1%	5	<1%	23	<1%
Total Number of Individuals	5110		4548		9658	

**Table 3.** List of macroinvertebrate taxa collected during 2006-2007 from the Patoka River, Muscatatuck, and Big Oaks National Wildlife Refuges. Numbers indicate the sites at which each species has been collected based on information in Supplemental materials Suppl. material 1 Appendix a, b, d and Figs 1, 2, 3.

Taxa List	Patoka River	Muscatatuck	Big Oaks
Ephemeroptera	67		
Ameletidae			
*Ameletus* spp. Eaton 1885	15, 18, 27		
Baetidae	23, 26, 38, 43, 44, 72		13, 15
*Acerpenna pygmaea* (Hagen 1861)	14, 15, 18, 25, 27, 30	8, 11	
*Baetis flavistriga* McDunnough 1921	4, 14		5
*Baetis intercalaris* McDunnough 1921	3, 19, 26, 34, 36		20
*Callibaetis* spp. Eaton 1881	23, 24, 26, 30, 35, 44, 72	16, 17	
*Centroptilum* spp. Eaton 1869	5, 8, 15-16, 17, 20, 27, 42-43, 47-48, 50		15, 29
*Plauditus dubius* (Walsh 1862)	14		
*Plauditus* spp. Lugo-Ortiz and McCafferty 1998	15, 18, 22, 27, 29	11	
*Procloeon* spp. Bengtsson 1915	19		
*Pseudocloeon* spp. Klapàlek 1905	26, 35, 78		
Caenidae			
*Caenis* spp. Stephens 1835	2-4, 7, 9, 14-18, 20, 22-23, 25-27, 29-31, 33, 38, 40-44, 47-48, 50-52, 55, 63, 65-66, 72-73	7, 8, 16	8-13, 15-16, 19-20, 25, 29, 30
Ephemeridae			
*Ephemera* spp. Linnaeus 1758			7, 10-12, 20, 25, 27, 30
*Ephemera simulans* Walker 1853			7-10, 12
*Hexagenia limbata* (Serville 1829)	26		11, 30
Ephemererellidae			
*Attenella attenuate* (McDunnough 1925)			8
*Eurylophella* spp. Tiensuu 1935		11, 12	
Heptageniidae	5, 34, 76, 77		8, 9, 12
*Nixe* spp. Flowers 1980	14, 15, 18, 22, 29, 37, 49, 82		
*Leucrocuta* spp. Flowers 1980			11, 20, 25
*Maccaffertium pulchellum* (Walsh 1862)			5, 11, 25
*Stenacron* spp. Jensen 1974		11	5, 11, 20, 27, 30
*Stenacron interpunctatum* (Say 1839)	7, 36, 42, 63, 76		9, 11
*Stenonema femoratum* (Say 1823)	1-5, 9, 14, 15, 18, 22-23, 25-26, 30, 42, 43	11	7-13, 15, 16, 20, 15, 29, 30
Isonychiidae			
*Isonychia* spp. Eaton 1871			12, 20, 25, 30
Leptohyphidae			
*Tricorythodes* spp. Ulmer 1920	6, 33-35, 63, 76-78		
Leptophlebiidae			
*Choroterpes* spp. Eaton 1881	5		20, 29
*Leptophlebia* spp. Westwood 1840		12, 17, 20	
*Paraleptophlebia* spp. Lestage 1917	1, 2, 10, 14, 15, 18, 22, 82, 83		7-12, 15
Siphlonuridae			
*Siphlonurus* spp. Eaton 1868	83	20	
Odonata	9, 20, 23, 38, 40, 43, 44, 67		
Calopterygidae	54		
*Calopteryx maculate* Beauvois 1805	39, 40, 44, 51, 63, 77		12
*Calopteryx* spp. Leach 1815	15, 18, 22, 30, 46, 48-50, 56, 59, 64, 66	8, 13, 19	
*Hetaerina americana* (Fabricius 1798)	13, 16, 26		
*Hetaerina* spp. Hagen 1853	40		
Coenagrionidae	1, 9, 14-15, 19, 20, 22-23, 25-27, 29-31, 33-35, 38, 40-41, 44, 47-48, 50, 52, 55-56, 59, 62, 66, 67, 72-73, 77, 82	13, 16, 17	7, 15
*Argia* spp Rambur 1842	6, 7, 11, 14, 16, 20, 25-26, 29, 30, 33, 40-44, 46-48, 50, 52-53, 56, 62, 66, 73, 76-78	13	9, 11, 12, 20
*Argia apicalis-tibialis* (Say 1839)	6, 7, 9-11, 20, 33, 34, 40, 54, 63, 76-78		
*Argia fumipennis* (Burmeister 1839)	9, 19, 23, 39, 40, 44, 51, 72		9
*Argia moesta* Selys 1865	76		9
*Argia sedula* (Hagen 1861)	26, 40		
*Enallagma basidens* Calvert 1902	9, 13, 16, 19, 23, 26, 38, 40, 72		13
*Enallagma divagans* Selys 1850	6, 13, 16, 20, 26, 33, 34, 38-40, 44, 72, 77		8, 10, 12, 13
*Enallagma exsulans* (Hagen 1861)	9, 13, 16, 19, 20, 23, 38, 40, 72		
*Enallagma signatum* (Hagen 1861)	67		
*Enallagma* spp. Charpentier 1840	2, 7, 13, 16, 19, 20, 23, 26, 38-40, 72		
*Ischnura posita* (Hagen 1861)	9, 33, 63		
*Ischnura posita-verticalis* (Hagen 1861)	1, 38		
*Ischnura* spp. Charpentier 1840	9, 17, 23, 24-25, 27, 33, 35, 38, 40, 63, 67, 72		10
Aeshnidae			
*Aeshna umbrosa* Walker 1908	35		
*Basiaeschna Janata* (Say 1839)	1-3, 13, 16, 20, 38-40, 42-44, 54, 77	13	13, 15
*Boyeria vinosa* (Say 1839)	1, 40, 44, 51		
*Boyeria* spp. McLachlan 1895	41, 46	13	12
*Nasiaeschna pentacantha* (Rambur 1842)	34, 77		
Cordulegastridae			
*Cordulegaster* spp. Leach 1815	49, 83		12, 32
Corduliidae			15
*Epitheca princeps* Hagen 1861	11, 16, 26, 33, 38, 43		
*Epitheca* spp Burmeister 1839	40, 43		
*Somatochlora ensigera* Martin 1906	43		
*Somatochlora* spp Selys 1871	42, 67		7, 15, 18, 27, 32
*Tetragoneuria* spp. Hagen 1861		16	
Libellulidae		17	
*Erythemis simplicicollis* (Say 1839)		17	
*Libellula* spp. Linnaeus 1758	14, 29, 56, 59, 62, 73		13, 19
*Libellula luctuosa* Burmeister 1839			15
*Pachydiplax longipennis* (Burmeister 1839)		7, 12	13
*Pantala hymenaea* (Say 1839)	23		
*Perithemis tenera* (Say 1839)	72		13
*Plathemis Lydia* (Drury 1773)	26, 38, 42, 72		13, 15
*Sympetrum* spp. Newman 1833	40		
Macromiidae			
*Didymops transversa* (Say 1839)			13
*Macromia taeniolata* Rambur 1842	78		
*Macromia* spp. Rambur 1842	7, 34, 39, 42, 43, 51, 55		
Gomphidae	54, 62		13, 30
*Dromogomphus spinosus* Selys 1854	38-40, 44		7, 9, 12, 15
*Dromogomphus spoliatus* (Hagen 1858)	1, 6, 42		
*Dromogomphus* spp. Selys 1854	11		
*Gomphus* spp. Leach 1815	64, 66		5, 11, 30
*Hagenius brevistylus* Selys 1854			11, 30
*Progomphus obscurus* (Rambur 1842)	44, 51, 64		10
Plecoptera			
Leuctridae			
*Leuctra* spp. Stephens 1835	3, 14, 15, 37, 48, 82		5
Nemouridae			
*Amphinemura* spp. Ris 1902	14, 18, 22, 29, 37, 41, 46-50, 82, 83, 86	11, 19	
Perlidae			
*Acroneuria* spp. Pictet 1841	3		5, 12, 30
*Acroneuria evoluta* Klapàlek 1909			9
*Neoperla* spp. Needham 1905	3		
Perlodidae	56		
*Isoperla* spp. Banks 1906	14, 15, 18, 22, 27, 29, 49, 82	11	
Hemiptera			
Belostomatidae			
*Belostoma flumineum* Say 1832	23, 26, 35, 38		13
*Belostoma lutarium* (Stàhl 1855)	30, 41, 62	17	
*Belostoma* spp. Latreille 1807	1, 34, 72		
Corixidae	1, 11, 23, 36, 38, 43, 67		15
*Palmacorixa nana* Walley 1930	11		
*Sigara modesta* (Abbott 1916)	15		16
*Sigara* spp. Fabricius 1775	17		15
*Trichocorixa calva* (Say 1832)	1, 20, 33, 36, 43, 55, 59, 67		15
*Trichocorixa kanza* Sailer 1948	77, 78		
*Trichocorixa* spp. Kirkaldy 1908	35, 76		
Gerridae	1, 2, 9, 23, 38		
Gerridae larvae	41		15
*Aquarius* spp. Schellenberg 1800	3, 8, 17		7
*Aquarius remigis* (Say 1832)			23
*Gerris* spp. Fabricius 1794	8, 17, 35, 38-40, 42, 67, 72		
*Limnoporus* spp. Stàhl 1868	72, 77		
*Rheumatobates palosi* Blatchley 1926	38		
*Rheumatobates rileyi* Bergroth 1892	3		
*Rheumatobates tenuipes* Meinert 1895	6		
*Rheumatobates* spp. Bergroth 1892	3, 6, 11, 13, 16, 20, 26, 33, 34, 36, 38, 39, 42, 43, 77, 78		
*Trepobates pictus* (Herrich-Schaeffer 1847)	4, 5, 42		
*Trepobates subnitidus* Esaki 1926	6, 9, 13, 16, 19, 20, 38, 72		13
*Trepobates* spp. Uhler 1883	3, 5, 7, 9, 13, 16, 19, 20, 26, 42, 72		29
Hebridae			
*Hebrus* spp. Curtis 1831	23		
Hydrometridae			
*Hydrometra martini* Kirkaldy 1900	1, 44		
Mesoveliidae			
*Mesovelia mulsanti* White 1879	1, 6, 11, 13, 16, 20, 38, 72		8
Naucoridae			
*Pelocoris femoratus* (Palisot 1820)	38, 67	17	
Nepidae			
*Ranatra buenoi* Hungerford 1922	11, 39		12
*Ranatra nigra* Herrich-Schaeffer 1849		16	
Notonectidae			
*Notonecta irrorata* Uhler 1879	43, 77, 83		13
*Notonecta* spp. Linnaeus 1758	24, 36		
Pleidae			
*Neoplea striola* (Fieber 1844)	9, 11, 13, 20, 33, 38, 67, 72		10
Saldidae			
*Micracanthia* spp. Reuter 1912	19		
Veliidae	23		
*Microvelia americana* (Uhler 1884)	1, 3-5, 8, 13, 17, 35, 39, 40, 42-44, 51, 77		
*Microvelia* spp. Westwood 1834	19, 23, 35, 43, 46-47, 54, 67, 72, 83-86		
*Rhagovelia obesa* Uhler 1871	39, 40		
*Rhagovelia* spp. Mayr 1865	76		
Megaloptera			
Corydalidae			
*Chauliodes pectinicornis* (Linnaeus 1763)	36		15
*Chauliodes* spp. Latreille 1796	70		
*Corydalus cornutus* (Linnaeus 1758)			5, 8, 11-13, 20, 25
*Nigronia* spp. Banks 1908			
*Nigronia serricornis* (Say 1824)	3		5, 7, 11-13, 15, 20, 25, 30
Sialidae			
*Sialis* spp. Latreille 1802	1-4, 38, 39, 42-44, 51, 53-54, 62, 83		7, 8, 10, 11-13, 15, 16, 27, 30
Trichoptera			
Hydropsychidae			
*Cheumatopsyche* spp. Wallengren 1891	3-5, 13-15, 19, 20, 25-27, 29-30, 33, 34, 36, 39, 41, 44, 47-52, 54-55, 63-65, 77-78	9, 11, 13	5, 8, 11, 12, 20
*Hydropsyche betteni* Ross 1938	25, 29, 50		5
*Hydropsyche betteni-depravata* Hagen 1861	19, 51		
*Hydropsyche cuanis* Ross 1938	36		
*Hydropsyche hageni* Banks 1905	63		
*Hydropsyche simulans* Ross 1938	4, 7, 33, 34, 54, 76, 77, 78		
*Hydropsyche* spp. pupae Pictet 1834	22	9	
Helicopsychidae			
*Helicopsyche borealis* (Hagen 1861)			7, 15, 25
Hydroptilidae			
*Hydroptila* spp. Dalman 1819	25, 27, 38, 40, 47, 48, 50, 53, 55, 59, 64		
*Oxyethira* spp. Eaton 1873	44		
Leptoceridae	20, 39		
*Ceraclea flava* (Banks 1904)			8, 9
*Nectopsyche candida* (Hagen 1861)	77		
*Nectopsyche exquisita* (Walker 1852)	34, 78		
*Nectopsyche* spp. Mueller 1879	26, 76, 78		
*Oecetis cinerascens* (Hagen 1861)	40		
*Oecetis* spp. McLachlan 1877	14, 20, 38, 40-41, 56, 64		
Limnephilidae			
*Ironoquia* spp. Banks 1916	18, 22, 27, 29, 41, 46, 49, 82, 84	8-10, 19, 20	
*Pycnopsyche* spp. Banks 1905		12	
Philopotamidae			
*Chimarra aterrima* Hagen 1861	4, 19		
*Chimarra obscura* (Walker 1852)	19, 51		
*Chimarra* spp. Stephens 1829	41, 47, 48, 50		5, 11, 20, 30
Phryganeidae			
*Ptilostomis* spp. Kolenati 1859	83, 85	17	
Polycentropodidae			
*Cernotina spicata* Ross 1938	2, 4		8, 10
*Neureclipsis crepuscularis* (Walker 1852)	5, 7, 34, 63, 76-78		
*Polycentropus* spp. Curtis 1835	83		
Rhyacophilidae			
*Rhyacophila* spp. Pictet 1834	27, 41	11, 19	
Uenoidae			
*Neophylax* spp. McLachlan 1871	15		
Lepidoptera			
Crambidae			8
*Acentria* spp. Stephens 1829	26		
Noctuidae			
*Bellura* spp. Walker 1865	8		
Pyralidae		17	19
Coleoptera			
Curculionidae	26, 34		7
Dryopidae			
*Helichus basalis* LeConte 1852	39		7, 8, 10, 12, 20, 25, 27, 30
*Helichus fastigiatus* (Say 1824)	41		
*Helichus lithophilus* (Germar 1824)	2, 9, 16, 18-20, 40, 44, 77		9, 12, 20, 25
Dytiscidae	44		
*Acilius* spp. larvae Leach 1817		20	
*Acilius fraternus* (Harris 1828)	43		18
*Agabus gagates* Aubè 1838			18
*Agabus semivittatus* LeConte 1852	84		
*Agabus* spp. Leach 1817	15, 18, 22, 29, 30, 47, 52		
*Brachyvatus apicatus* (Clark 1862)	72		
*Copelatus chevrolati* Aubè 1838	67		
*Copelatus glyphicus* (Say 1823)	35		
*Heterosternuta laetus* (Leech 1948)	82		
*Heterosternuta pulcher* (LeConte 1855)	22, 29		
*Hydroporus* spp. Clairville 1806	17, 44		
*Laccophilus fasciatus* Aubè 1838	24, 35, 67		27
*Laccophilus* spp. Leach 1815	23, 24, 35		
*Laccophilus maculosus* Say 1823			13
*Liodessus* spp. Guignot 1939	23		
(Hydroporinae)	18, 22, 29, 62, 66	16	
*Neoporus dimidiatus* (Gemminger and Harold 1868)	55, 65, 66		
*Neoporus* spp. Guignot 1931	1-3, 11, 16, 23, 26, 34, 38, 40, 42, 43, 78		7, 9, 11-13, 15
*Neoporus undulatas* (Say 1823)	25, 73, 83	12, 16	
Elmidae			
*Ancyronyx variegatus* (Germar 1824)	1, 2, 7, 33, 34, 38, 76-78		
*Dubiraphia minima* Hilsenhoff 1973	1-3, 6, 7, 9, 16, 20, 23, 26, 33, 34, 38-40, 43, 44, 54, 63, 76-78		8-10, 13, 15
*Dubiraphia quadrinotata* (Say 1825)	77		
*Dubiraphia* spp. Sanderson 1954	23, 26, 38-40, 43, 44		8, 9, 13, 15
*Dubiraphia* spp. larvae and adults Sanderson 1954	14, 47, 48, 52, 46, 59, 64-66, 70, 73		
*Macronychus glabratus* Say 1825	7, 11, 33, 34, 38-40, 63, 76-78		10
*Stenelmis crenata* (Say 1824)	2-4, 6, 11, 16, 20, 26, 33, 34, 36, 38-40, 51, 63, 76-78		
*Stenelmis decorata* Sanderson 1938	51		
*Stenelmis quadrimaculata* Horn 1870	44		
*Stenelmis sexlineata* Sanderson 1938			8-10
*Stenelmis* spp. Dufour 1835	3-5, 19, 26, 34, 40, 44, 76		5, 8-10, 13, 20, 25, 29
*Stenelmis* spp. larvae and adults Dufour 1835	14, 18, 22, 25, 27, 29, 30, 41, 46-50, 55, 56, 64		
Gyrinidae	44		
*Dineutus serrulatus* LeConte 1868	10, 43		
*Dineutus* spp. Macleay 1825	20		
*Dineutus* spp. adults Macleay 1825	73	13	
*Gyretes sinuatus* Leconte 1851	6, 77		
*Gyrinus* spp. Geoffroy 1762	35, 42-44		
*Gyrinus* spp. adults Geoffroy 1762	47, 55		
Haliplidae			
*Haliplus deceptus* Matheson 1912	62		
*Peltodytes dunavani* Young 1961	38, 40, 86	9	
*Peltodytes duodecimpunctatus*	1, 9, 11, 13, 15-18, 20, 23, 26, 29-31, 35, 37-38, 40, 43, 44, 47-48, 50, 55, 59, 66-67, 72-73	13, 16	8, 12-13, 15, 16, 23, 27, 29
*Peltodytes edentulus* (LeConte 1863)	23		
*Peltodytes lengi* Roberts 1913	1, 2, 5, 16, 20, 23, 26, 33, 35, 39, 40, 44, 67		8, 13, 15
*Peltodytes litoralis* Matheson 1912	31, 44		
*Peltodytes muticus* (LeConte 1853)	1, 48, 62, 67, 70	12, 17, 20	15
*Peltodytes pedunculatus* Blatchley 1910	1		13
*Peltodytes sexmaculatus* Roberts 1913	16, 20, 35, 59, 65, 67		13
*Peltodytes* spp. Règimbart 1878	20, 35, 44, 67		
*Peltodytes* spp. larvae Règimbart 1878	59, 62, 66	17	
Heteroceridae	13, 23, 35, 39, 40, 72		
Hydrochidae			
*Hydrochus* spp. Leach 1817	1, 43, 67		
Hydrophilidae	35, 72		
*Berosus infuscatus* LeConte 1855	2, 67		
*Berosus peregrinus* (Herbst 1797)	9, 13, 16, 20, 23, 26, 35, 38, 40, 44, 51, 72		15
*Berosus* spp. Leach 1817	9, 16, 20, 23, 26, 34, 38, 40, 44, 51, 72		13. 15
*Berosus* spp. larvae Leach 1817	14, 18, 25, 29, 46, 48, 50, 53, 55, 56, 59, 62, 64, 73		
*Enochrus pygmaeus nebulosus* (Say 1824)	22		
*Enochrus* spp. Thomson 1859	1, 13, 24, 26, 35, 40, 44		
*Hydrobius* spp. Leach 1815	35		
*Hydrochara* spp. larvae Berthold 1827		10	
*Hydrochara soror* Smetana 1980		20	
*Paracymus* spp. Thomson 1867	8, 17, 35		
*Tropisternus glaber* (Herbst 1797)	23, 35, 40, 44, 67		8, 15
*Tropisternus lateralis nimbatus* (Say 1823)	67		
*Tropisternus natator* Orchymont 1938			27
*Tropisternus* spp. Solier 1834	16, 17, 23, 24, 26, 39, 44		
*Tropisternus* spp. larvae Solier 1834	27		
Psephenidae			
*Ectopria* spp. larvae LeConte 1853			11, 23
*Psephenus herricki* (DeKay 1844)	3-5, 13		5, 7-12, 18, 20, 23, 25, 29, 30
Scirtidae			
*Cyphon* spp. Paykull	1, 3, 5, 6, 9, 10, 23, 26, 33		10, 12, 13
*Scirtes* spp. Illiger	46, 70	20	
Diptera			
Chironomidae	1, 3, 4, 11, 13, 23, 26, 33-35, 38, 40, 43, 44, 72		7, 15
(Diamesinae)			
*Diamesa* spp.	14, 15, 18		
*Potthastia longimana* group (Keiffer 1922)	29		
(Orthocladiinae)	7		
*Acricotopus* spp.	62		
*Chaetocladius* spp. Kieffer	15, 49, 55, 82, 84-86		
*Corynoneura* spp.		9	7, 8, 15
*Cricotopus* spp. Wulp 1874		7-9, 11, 13, 16	
*Hydrobaenus* spp. Fries		8, 9, 11, 13	
*Limnophyes* spp. Eaton	15, 18, 22, 46, 47, 50		
*Nanocladius* spp. Keiffer		7	20
*Orthocladius* spp. Wulp 1874	23, 26, 35, 40		
*Parametriocnemus* spp. Goetghebuer 1932	18, 41		
*Paraphaenocladius* spp.	44		
*Pseudorthocladius* spp.	85		
*Smittia* spp.	31		
(Corynoneurini)			
*Corynoneura* spp.	15, 18, 22, 49, 59		
*Thienemanniella* spp. Kieffer	14, 22, 30, 48, 49, 52, 56, 73		
(Orthocladiini/Metriocnemini)			
*Cricotopus* spp. Wulp 1874	14, 15, 18, 22, 25, 27, 29, 30, 31, 37, 41, 46-50, 52, 53, 55, 56, 59, 62, 64-66, 70, 73, 82, 84		
*Cricotopus sylvestris* (Fabricius 1794)	38		
*Cricotopus/Orthocladius* spp. Wulp 1874	26, 35, 44		
*Eukiefferiella* spp. Thienemann	49		
*Hydrobaenus* spp. Fries	15, 18, 22, 47-50, 52, 55, 56, 59, 64, 66, 73		
*Nanocladius* spp. Keiffer	52, 72		
*Parakiefferiella* spp.	47, 62		
*Psectrocladius* spp. Kieffer	62		
*Rheocricotopus* spp. Thienemann and Harnisch	82		
(Chironominae)	5, 6, 8, 13, 17, 20, 26, 33, 35, 43, 72, 77		15
(Chironomini)	5, 8, 14-5, 19, 23, 25, 27, 33, 35, 38-40, 43, 56, 62, 64, 70		7, 10-12
*Chironomus* spp.	1, 8, 15, 22, 24, 29, 34, 35, 39, 43, 44, 52, 53, 56, 58, 59, 62, 64, 66, 70, 73, 86	9, 17, 20	10-11, 18
*Cladopelma* spp. Kieffer 1921	62		
*Cryptochironomus* spp.	6, 23, 25-26, 35, 38, 40, 42, 43, 50, 56, 64, 66, 73		
*Cryptotendipes* spp. Lenz	26, 38, 40, 43, 56, 66		
*Dicrotendipes* spp. Kieffer 1913	5, 9, 13, 15-16, 19, 20, 23, 25-27, 29-30, 35, 38, 40, 42-44, 56, 62, 66, 72-73	7, 11	29
*Endochironomus* spp. Kieffer	7, 16, 35, 52, 62, 73	16, 17	
*Glyptotendipes* spp.	42, 43, 63, 70, 86	7, 11, 16, 17	9, 10, 12, 15, 30
*Microtendipes* spp.	4, 5, 8, 22, 44		5, 7, 9, 10, 12, 20
*Parachironomus* spp. Lenz	6, 7, 16, 52	16	
*Paralauterborniella* spp. Lenz 1941	39, 40, 43, 70, 76		
*Paratendipes* spp.	15, 18, 29, 30, 42, 47, 70	11	11, 23
*Phaenopsectra* spp. Kieffer	5, 11, 13, 19, 35, 39, 42, 56		
*Polypedilum fallax* (Johannsen 1905)	13		
*Polypedilum* spp. Kieffer 1913	1, 2, 4-7, 9, 13-16, 18-20, 22-23, 25-27, 29-31, 33-36, 38-44, 46-49, 51-52, 55-56, 63-64, 66-67, 72-73, 77-78, 83	7-9, 11, 13, 16, 17	5, 7, 10-11, 13, 20, 30
*Saetheria tylus* (Townes 1945)		8	
*Stenochironomus* spp.	50		
*Stictochironomus* spp. Kieffer	3, 4, 26, 42, 43		7-8, 10-12, 15-16, 27, 29, 32
*Tribelos* spp.	77		
(Pseudochironomini)			
*Pseudochironomus* spp.	22-23, 25-26, 29-30		
(Tanytarsini)	9, 33, 35, 43, 44, 63		15
*Cladotanytarsus* spp. Kieffer 1921	9, 18, 20, 22-23, 26, 43, 44, 48, 50, 64, 67	11	10, 11, 13
*Micropsectra* spp. Kieffer 1909	15, 18, 22, 29, 48, 49, 52, 53, 59, 62, 66	9	
*Micropsectra/Tanytarsus* spp. Kieffer 1909	3, 8, 13, 20, 23, 26, 34, 35, 38, 40, 43, 44,		
*Paratanytarsus* spp. Bause	8, 14, 26-27, 29, 35, 38, 40, 44, 59, 62, 64, 66, 70	7	
*Rheotanytarsus* spp. Bause and Thienemann 1913	14, 25-27, 30, 33-34, 40, 43, 46, 49, 50, 52, 70		5
*Stempellinella* spp. Brundin			9
*Tanytarsus* spp. Van Der Wulp	14-16, 18, 20, 22, 25-27, 30, 35, 38, 40-41, 43-44, 47, 48, 50-51, 55, 56, 59, 62, 64, 66, 70, 83	7, 10	11, 13, 15, 29, 30
(Tanypodinae)	4, 18, 35		15
(Coelotanypodini)			
*Clinotanypus* spp.	20, 23, 40-41, 72	12	
(Natarsiini)			
*Natarsia baltimoreus* (Macquart 1855)	1, 4, 17, 42, 44		7
*Natarsia* spp.	22, 29, 79		
(Procladiini)			
*Procladius* spp. Skuse 1889	16, 20, 35, 38-40, 42, 43, 52, 55, 59, 62, 72	16	11
(Pentanuerini)			
*Ablabesmyia janta* (Roback 1959)	2, 6, 7, 11, 33, 34, 63, 76-78		
*Ablabesmyia mallochi* (Walley 1925)	3, 5, 7, 17, 26, 33, 35, 38-40, 42-44, 72		
*Ablabesmyia* spp. Johannsen 1905	15, 48, 50, 53	16, 17	
*Labrundinia becki* Roback 1971	1		
*Labrundinia pilosella* (Loew 1866)	33, 34, 43, 63		
*Labrundinia* spp.	40	17	
*Larsia* spp. Wiedemann 1824	5, 18, 22, 31, 40, 46, 52, 62, 64, 70, 73, 79, 82	16	
*Paramerina* spp.	35		
*Pentaneura* spp.	66		
*Thienemannimyia* group	1, 5, 13-15, 17-19, 22, 25-27, 29-31, 33, 35, 40, 46-48, 50-51, 55-56, 64, 66	8, 9	8
*Zavrelimyia* spp. Kittkau	15, 37, 48, 79, 82		
(Tanypodini)			
*Tanypus neopunctipennis* Sublette 1964	67, 72		
*Tanypus* spp. Meigen 1803	59, 62	17	
Ceratopogonidae	47, 50, 53-55, 56, 59, 62, 64, 66, 70, 85		19
*Atrichopogon* spp.	13, 24		
*Bezzia-Palpomyia* group	2, 3, 40, 46, 70		
*Ceratopogon* spp. Meigen 1803	33, 35, 38, 40, 43, 54		
*Dasyhelea* spp.	44, 53, 59, 62		
*Forcipomyia* spp. Meigen 1818	23		
*Probezzia* spp. Kieffer 1906	26		
*Serromyia* spp.	40		
Chaoboridae			
*Chaoborus* spp. Lichtenstein 1800	18		
Culicidae			
*Aedes* Meigen 1818 */ Ochlerotatus*	86		
*Aedes* spp. Meigen 1818	24		
*Anopheles punctipennis* (Say 1823)	4, 6, 8, 9, 17, 36		
*Anopheles quadrimaculatus* Say 1824	67		
*Anopheles* spp. Meigen 1818	24, 42, 44		
Dixidae			12
*Dixella* spp.	3, 4, 6, 7		
Dolichopodidae	31		
Empididae			
*Clinocera* spp. Meigen 1803	14		
*Hemerodromia* spp.	3, 4, 14, 44, 47, 48, 50, 56, 64, 66		
Ephydridae	27, 59		
Ptychopteridae			
*Bittacomorpha* spp. Westwood 1835			18
Sciomyzidae			7, 9
Simuliidae			
*Simulium jenningsi* Malloch 1914	4		
*Simulium tuberosum* (Lundstrom 1911)	3		
*Simulium vittatum* (Zetterstedt 1838)	51		
*Simulium* spp. Latreille 1802	25, 27, 29, 46, 52, 53, 64-66, 70, 73	7, 11	
Stratiomyidae			
*Stratiomys* spp. Geoffroy 1762	2, 67		7, 13
Tabanidae	29, 37, 41, 50, 53, 62, 83	9	
*Chlorotabanus crepuscularis* Bequaert 1926	1, 42		
*Chrysops* spp.	1, 6, 23, 35, 40, 43, 44		13
*Tabanus* spp.	72		8
Tipulidae	26		7
*Hexatoma* spp. Latreille 1809			5, 7-8, 12, 20
*Limnophila* spp. Macquart 1834			9
*Pilaria* spp. Sintenis 1889	49		11, 12
*Pseudolimnophila* spp.	4		
*Tipula* spp. Linnaeus 1758	14, 18-19, 29, 30, 38, 41, 56, 64, 82	11, 13	8
Tipulidae pupae	47		
Orthoptera			
Acrididae			
*Metaleptea brevicornis* (Johannson 1763)			13
Collembola			
Isotomidae	4, 10, 20, 24, 26, 35, 38, 67, 72		9
Poduridae	63		
Sminthuridae	23		
Decapoda			
Cambaridae			
*Cambarus* species “A”	24, 35, 44, 48, 54, 67	7, 19-20	18
*Cambarus polychromatus* Thoma, Jezerinac, and Simon 2005	35, 39, 44		
*Orconectes immunis* (Hagen 1870)	11, 14-15, 24, 29, 30, 34, 35, 37, 56, 65, 70		
*Orconectes indianensis* (Hay 1896)	1-3, 6, 7, 17, 19, 22, 25-26, 29, 34, 38-40		
*Orconectes juvenilis* Taylor 2002			9-12
*Orconectes juvenilis x virilis*			12
*Orconectes propinquus* (Girard 1852)	63		
*Orconectes rusticus* (Girard 1852)			9, 11
*Orconectes sloanii* (Bundy 1876)			5, 7-13, 15, 16, 20, 23, 25, 27, 29, 30, 32
*Orconectes virilis* (Hagen 1870)	5		13
*Procambarus acutus* (Girard 1852)	59		
*Procambarus* spp. Ortmann 1905	62		
(Cambarinae)	14, 15, 22, 25, 27, 29-31, 37, 41, 47-50, 55, 56, 65, 66, 73, 79, 83, 84		
Amphipoda			
Hyalellidae			
*Hyalella azteca* Saussure 1858	1, 2, 18, 25-27, 38, 40, 46-48, 50, 52, 55, 59, 62-63	7, 12, 16, 17, 19	8, 9, 12, 13, 15
Crangonyctidae			
*Crangonyx* spp. Bate 1859	11, 18, 22, 27, 31, 37, 40-41, 46-50, 55, 63, 70, 73, 79, 82-84	8-10, 12, 13, 16, 19, 20	
*Synurella dentata* Hubricht 1943	22, 27, 29, 41, 64-66, 82, 83	8-11, 13, 19, 20	29
Isopoda			
Asellidae			
*Caecidotea* spp. Packard 1871	5, 14-15, 17-18, 22, 25, 27, 29-30, 35-37, 39, 40-43, 46-50, 52, 53, 56, 58-59, 62-66, 70, 73, 76, 82-84, 86	9, 10, 12, 13, 20	
*Lirceus fontinalis* Rafinesque 1820	1, 3, 22, 25, 27, 29-31, 35, 36, 73, 79	7-13, 16, 19, 20	13, 15, 20
Acariformes	4, 11, 35, 67		13, 15
Veneroida			
Corbiculidae			
*Corbicula fluminea* (Müller 1774)	26, 33, 34, 38-40, 44, 78		8, 11
*Corbicula* spp. Mühlfeld 1811	27, 56, 59, 64, 66, 73	13	12
Pisidiidae	65, 66		
*Pisidium* spp. Pfeiffer 1821	15, 25, 27, 29-31, 37, 58, 64-65, 67, 70	12	10, 18, 19, 27, 29
*Sphaerium* spp. Scopoli 1777	1, 6, 11, 33, 34, 38, 40, 42, 43, 56, 58, 59, 65, 67, 72, 76-78	7, 12, 16, 17, 19	5, 7-13, 15, 20, 25, 30, 32
Gastropoda			
Ancylidae			7
*Ferrissia* spp. Walker 1903	35		10
*Laevapex* spp. Walker 1903	43		
Lymnaeidae	22, 30, 31, 35, 37, 47, 48, 56, 59, 62, 73	9, 11-13, 20	
*Fossaria* spp. Westerlund 1885	35, 44, 67		
Planorbidae	35, 38, 40		
*Gyraulus* spp. Agassiz 1837	70	7, 12, 16	
*Helisoma* spp. Swainson 1840	1, 25, 31, 59, 64, 70	11	13, 15, 27, 29, 30
*Planorbella* spp.Haldeman 1842	26		13
Physidae			
*Physa* Draparnaud 1801*/Physella* spp. Haldeman 1842	1, 8, 9, 13, 15-17, 19- 20, 23-27, 30, 31, 33, 35, 37-44, 53, 56, 58-59, 62, 65, 67, 70, 72-73, 76-78	7, 9-13, 16, 20	7, 13, 15, 16, 18, 20, 23, 27, 32
Pleuroceridae			
*Elimia* spp. Adams and Adams 1854	1		5, 7-13, 15, 20, 25, 30
Viviparidae	40		
*Campeloma* spp. Rafinesque 1819	1		
Oligochaeta			8-13, 15
Tubificidae without hair chaetae			11, 13, 19
Branchiobdellida	35		7, 10, 11, 13
Enchytraeidae	14, 31, 55, 62		
Lumbricidae	15, 25, 30, 37, 47, 58, 73, 79, 83, 84, 86		18
Lumbriculidae	1, 10, 76, 79		
Naididae	16, 23, 24, 26, 34, 35, 38-40, 42, 43, 54, 67, 72, 76		13
*Chaetogaster diaphanus* (Gruithuisen 1828)		9, 16	
*Chaetogaster limnaei* Von Baer 1827			13
*Dero* spp. Okem 1815	58, 62, 66	7, 12, 16	
*Nais communis* Piquet 1906	14, 15, 30, 31, 58, 70, 83	9, 12, 13, 16, 19, 20	
*Nais pardalis* Piquet 1906	14, 22, 27, 29-31, 64, 66	13, 16	
*Nais variabilis* Piquet 1906	14, 30, 52, 53, 56, 58, 64, 73		25
*Pristina* spp. Ehrenberg 1828			16
*Pristina leidyi* Smith 1896	62		
*Ophidonais serpentine* (Mueller 1773)		7	
*Slavina appendiculata* (D’udekem 1855)	22	9, 16	16
*Stephensoniana tandyi* Harman 1975		7	
Tubificidae	34, 42, 51, 78		
*Branchiura sowerbyi* Beddard 1892	7, 11, 22, 29, 30, 43, 76		
*Limnodrilus* spp. Claparede 1862	30, 73		
*Potamothrix bavaricus* (Oschman 1913)	50		
*Tubifex tubifex* (Mueller 1774)	62		
*Quistradrilus multisetosus* Brinkhurst and Cook 1966		16	
Tubificidae with hair chaetae	22, 27, 29, 31, 37, 47, 56, 58, 59, 62, 64-66, 70	12	
Tubificidae without hair chaetae	14, 15, 18, 22, 25, 27, 29-31, 37, 41, 49, 50, 52, 55, 56, 58, 59, 62, 64-66, 70, 73, 83	12, 16, 19	
Hirudinea			
Erpobdellidae	35, 38, 43		
*Erpobdella punctate* (Leidy 1870)	14, 31	9	
*Mooreobdella fervida* (Verrill 1872)	58	20	
*Mooreobdella microstoma* (Moore 1901)			
Glossiphoniidae	33, 38, 43, 67	12	
Nematomorpha			
*Paragordius varius* (Leidy 1851)	11, 67		
Nemertea			
*Prostoma* spp. Duges 1828	52, 59		
Neuroptera			
*Climacia areolaris* (Hagen 1861)	33, 77, 78		
Turbellaria	6, 15, 19, 72, 83		
Planariidae			
*Dugesia* spp.	29, 52	7, 16	
Cnidaria			
*Hydra* spp. Linnaeus 1758		7, 16	

**Table 4.** List of crayfish taxa collected from three National Wildlife Refuges. Numbers indicate the sites at which each species has been collected based on information in Supplemental materials Appendix a-d and Figures 1-3.

**Species**	**Patoka**	**Muscatatuck**	**Big Oaks**
*Procambarus (Ortmannicus) acutus* (Girard 1852)	59, 62	13	
*Orconectes (Faxonius) indianensis* (Hay 1896)	1-3, 6-7, 17, 19, 22, 25, 26, 29, 34, 38-40, 63		
*O. (Gremicambarus) virilis* (Hagen 1870)	5		1, 2
*O. (Procericambarus) juvenilis* (Hagen 1870)			8-11, 15
*O. (Rhoadesius) sloanii* (Bundy 1876)		5, 6, 10, 13-15, 20	3-5, 7- 13, 15-17, 20-27, 29-33
*O. (Trisellescens) immunis* (Hagen 1870)	11, 14-15, 22, 24-25, 27, 29-31, 34-35, 37, 41, 47-50, 55-56, 65- 66, 70, 73, 79, 83, 84	6, 8-11, 13, 21	5
*Cambarus (Cambarus) ortmanni* Williamson 1907			10, 15, 19, 32
*C. (Erebicambarus) laevis* Faxon 1914		19	
*C. (Lacunicambarus)* species "A"	24, 35, 44, 48, 54, 67	1, 5, 7, 8-13, 15-16, 18-20	7, 10, 11, 18
*C. (Tubericambarus) polychromatus* Thoma, Jezerinac, and Simon 2005	35, 39, 44	8, 13, 15	3, 7, 10-11, 25, 33

**Table 5.** Comparison of fish assemblage structure and catch percentages from the Big Oaks National Wildlife Refuge.

	**2006**		**2007**		**Total**	
**Species**	**Count**	**%**	**Count**	**%**	**Count**	**%**
Cyprinidae						
*Campostoma anomalum*	426	8%	751	17%	1177	12%
*Cyprinella spiloptera*	10	<1%	18	<1%	28	<1%
*Ericymba buccata*	40	1%	171	4%	211	2%
*Hybopsis amblops*	72	1%	223	5%	295	3%
*Luxilus chrysocephalus*	182	3%	461	10%	643	7%
*Lythrurus umbratilis*	29	1%	16	<1%	45	<1%
*Notemigonus crysoleucas*	18	<1%			18	<1%
*Notropis ariommus*	10	<1%			10	<1%
*Notropis boops*	38	1%	28	1%	66	1%
*Notropis photogenis*			15	<1%	15	<1%
*Pimephales notatus*	964	18%	857	19%	1821	0
*Semotilus atromaculatus*	523	10%	485	11%	1008	10%
Esocidae						
*Esox americanus*	4	<1%			4	<1%
Catostomidae						
*Catostomus commersonii*	26	<1%	235	5%	261	3%
*Erimyzon oblongus*	30	1%	1	<1%	31	<1%
*Hypentelium nigricans*	39	1%	93	2%	132	1%
*Moxostoma duquesnei*	10	<1%	75	2%	85	1%
*Moxostoma erythrurum*	21	<1%	7	<1%	28	<1%
Ictaluridae						
*Ameiurus natalis*	18	<1%	8	<1%	26	<1%
*Ameiurus nebulosus*	1	<1%			1	<1%
*Noturus miurus*	6	<1%	3	<1%	9	<1%
Fundulidae						
*Fundulus notatus*	1	<1%			1	<1%
Poeciliidae						
*Gambusia affinis affinis*	123	2%	52	1%	175	2%
Atherinopsidae						
*Labidesthes sicculus*	2	<1%			2	<1%
Centrarchidae						
*Ambloplites rupestris*	12	<1%	6	<1%	18	<1%
*Lepomis cyanellus*	285	5%	78	2%	363	4%
*Lepomis macrochirus*	1159	22%	3	<1%	1162	12%
*Lepomis megalotis*	147	3%	148	3%	295	3%
*Lepomis microlophus*	161	3%			161	2%
*Micropterus dolomieu*	1	<1%			1	<1%
*Micropterus salmoides*	23	<1%	2	<1%	25	<1%
*Pomoxis nigromaculatus*	21	<1%			21	<1%
Percidae						
*Etheostoma blennioides*	72	1%	32	1%	104	1%
*Etheostoma caeruleum*	286	5%	137	3%	423	4%
*Etheostoma flabellare*	86	2%	66	1%	152	2%
*Etheostoma nigrum*	177	3%	375	8%	552	6%
*Etheostoma spectabile*	269	5%	109	2%	378	4%
Total Number of Individuals	5292		4455		9747	

**Table 6.** Biological diversity and integrity comparison of aquatic faunal assemblages at three National Wildlife Refuges in southern Indiana.

	**Species Richness**				**Mean IBI**		
Refuge	Macroinvertebrates	Crayfish	Fish	Total	2006	2007	Range
Patoka River NWR	355	6	82	443	35	31	0-56
Muscatatuck NWR	96	6	51	153	--	40	12-54
Big Oaks NWR	163	7	37	207	35	41	26-54
