# Supplementary material for: Biological Diversity, Ecological Health and Condition of Aquatic Assemblages at National Wildlife Refuges in Southern Indiana, USA
Source: Biodivers Data J. 2015 Jan 12;(3):e4300. doi: 10.3897/BDJ.3.e4300 (PMC4304268; doi:10.3897/BDJ.3.e4300)
Supplement: Supplementary material 1 — Supplemental Materials Appendix: Sites [file biodiversity_data_journal-3-e4300-s001.docx]

**Supplemental materials Appendix a.** Site locations sampled during an investigation of the Patoka River drainage.  Site numbers correspond to Table 1, 2, 3, and 4 and are shown in Figure 1.

| Site No | County | Locality | Latitude | Longitude |
| --- | --- | --- | --- | --- |
| 1 | Orange | Patoka River @ CR 50 W | 38º 26.84' | -86º 28.19' |
| 2 | Orange | Patoka River @ CR 175 W | 38º 26.45' | -86º 29' |
| 3 | Orange | Youngs Creek @ CR 350 W | 38º 28.24' | -86º 29.87' |
| 4 | Crawford | Ritter Creek @ Dillard Rd | 38º 21.7' | -86º 39.48' |
| 5 | Crawford | Ritter Creek @ SR 164 | 38º 22.08' | -86º 39.92' |
| 6 | Dubois | Patoka River @ NE Dubois Rd | 38º 28.23' | -86º 45.71' |
| 7 | Dubois | Patoka River @ NE Dubois Rd | 38º 28.33' | -86º 46.64' |
| 8 | Dubois | Leistner Creek @ SR 56 | 38º 28.88' | -86º 49.07' |
| 9 | Dubois | Polson Creek @ SR 545 | 38º 26.31' | -86º 48.4' |
| 10 | Dubois | Patoka River @ CR 175 E | 38º 25.69' | -86º 52.22' |
| 11 | Dubois | Patoka River @ Patoka River Walk | 38º 23.81' | -86º 54.87' |
| 12 | Dubois | Patoka River @ SR 164/162 Bridge | 38º 23.26' | -86º 55.62' |
| 13 | Dubois | Hall Creek @ CR 100 S | 38º 21.87' | -86º 46.6' |
| 14 | Dubois | Hall Creek Trib @ Celestine Rd S | 38º 21.85' | -86º 46.99' |
| 15 | Dubois | Hall Creek Trib @ Celestine Rd | 38º 20.89' | -86º 46.74' |
| 16 | Dubois | Hall Creek @ SR 162 | 38º 21.39' | -86º 53.15' |
| 17 | Dubois | Unnamed Trib of Flat Creek @ CR 450 S | 38º 18.82' | -86º 48.63' |
| 18 | Dubois | Flat Creek Trib @ St. Anthony Rd Bridge | 38º 18.43' | -86º 50.22' |
| 19 | Dubois | Flat Creek @ CR 450 S | 38º 19.05' | -86º 50.79' |
| 20 | Dubois | Hall Creek @ SR 162 | 38º 21.21' | -86º 54' |
| 21 | Dubois | Trib Green Creek @ U/S CR 350 E Bridge | 38º 14.54' | -86º 50.28' |
| 22 | Dubois | Green Creek @ U/S SR 264 Bridge | 38º 14.49' | -86º 50.58' |
| 23 | Dubois | Green Creek @ Holiday Lake Rd | 38º 14.61' | -86º 52.17' |
| 24 | Spencer | Unnamed Trib of Hunley Creek @ E of CR 600 E | 38º 11.38' | -86º 56.09' |
| 25 | Dubois | Hunley Creek @ U/S CR 1000 S Bridge | 38º 14.02' | -86º 53.81' |
| 26 | Dubois | Hunley Creek @ CR 850 S | 38º 15.18' | -86º 54.15' |
| 27 | Dubois | Hunley Creek @ U/S CR E 660 S Bridge | 38º 17.03' | -86º 54.24' |
| 28 | Dubois | Indian Creek @ U/S W Old Road 64 Bridge | 38º 18.09' | -86º 55.35' |
| 29 | Dubois | Short Creek Trib @ U/S CR 900 S | 38º 14.82' | -86º 58.81' |
| 30 | Dubois | Short Creek @ U/S CR 400 W Bridge | 38º 15.67' | -86º 58.77' |
| 31 | Dubois | Hunley Creek Trib @ U/S Cherry St Bridge | 38º 18.81' | -86º 56.54' |
| 32 | Dubois | Hunley Creek @ U/S SR 231 Bridge | 38º 20.35' | -86º 56.95' |
| 33 | Dubois | Patoka River @ Ell Creek Rd | 38º 20.13' | -86º 59.41' |
| 34 | Dubois | Patoka River @ Ell Creek Rd | 38º 20.65' | -86º 59.8' |
| 35 | Dubois | Altar Creek @ SR 56 | 38º 25.07' | -87º 0.13' |
| 36 | Dubois | Patoka River @ CR 100 S | 38º 21.97' | -87º 2.41' |
| 37 | Dubois | Patoka River Trib @ U/S CR 100 S Bridge | 38º 21.85' | -87º 3.25' |
| 38 | Pike | Flat Creek @ CR 900 N | 38º 24.24' | -87º 4.59' |
| 39 | Pike | Flat Creek @ CR 250 N | 38º 27.6' | -87º 7.92' |
| 40 | Pike | Flat Creek @ CR 700 E | 38º 28.08' | -87º 9.05' |
| 41 | Pike | Unnamed Trib of Patoka River @ U/S CR 200 S | 38º 23.44' | -87º 11.03' |
| 42 | Pike | Rock Creek @ CR 1025 E | 38º 19.12' | -87º 5.34' |
| 43 | Pike | Rock Creek @ CR 925 E | 38º 19.84' | -87º 6.14' |
| 44 | Pike | Beadens Creek @ CR 775 E | 38º 19.11' | -87º 7.67' |
| 45 | Pike | Cup Creek @ U/S CR 625 S Bridge | 38º 19.86' | -87º 7.46' |
| 46 | Warrick | South Fork Patoka River @ U/S CR 1300 S Bridge | 38º 13.89' | -87º 10.77' |
| 47 | Pike | South Fork Patoka River @ U/S CR 1200 S Bridge | 38º 14.74' | -87º 11.37' |
| 48 | Pike | South Fork Patoka River @ U/S CR 1100 S Bridge | 38º 15.6' | -87º 11.93' |
| 49 | Pike | South Fork Patoka River Trib @ U/S CR 550 E | 38º 16.91' | -87º 10.32' |
| 50 | Pike | South Fork Patoka River @ U/S CR 300 E | 38º 17.23' | -87º 13.16' |
| 51 | Pike | South Fork Patoka River @ CR 875 S | 38º 17.38' | -87º 13.47' |
| 52 | Pike | Rough Creek @ U/S CR 1200 S | 38º 14.74' | -87º 15.12' |
| 53 | Pike | Rough Creek @ U/S CR 925 S | 38º 17.14' | -87º 14.56' |
| 54 | Pike | South Fork Patoka River @ CR 900 S | 38º 17.68' | -87º 14.87' |
| 55 | Pike | South Fork Patoka River @ U/S State Hwy 61 | 38º 17.83' | -87º 15.64' |
| 56 | Pike | Honey Creek @ U/S CR 900 S | 38º 17.37' | -87º 15.94' |
| 57 | Pike | Hat Creek @ U/S CR 50 E | 38º 18.69' | -87º 16.19' |
| 58 | Gibson | Turkey Creek @ CR 1275 E | 38º 20.3' | -87º 19.83' |
| 59 | Gibson | Unnamed Trib of Turkey Creek @ CR 75 S | 38º 20.57' | -87º 19.37' |
| 60 | Gibson | South Fork Patoka River @ SR 57 Bridge | 38º 22.68' | -87º 20.21' |
| 61 | Pike | Patoka River @ U/S SR 61 Bridge | 38º 22.8' | -87º 13.03' |
| 62 | Pike | Stone Coe Creek @ U/S SR 61 Bridge | 38º 23.69' | -87º 13.19' |
| 63 | Pike | Patoka River @ Meridian Rd | 38º 23.01' | -87º 16.14' |
| 64 | Pike | Flat Creek @ Chandler Rd Bridge | 38º 25.29' | -87º 18.06' |
| 65 | Pike | Flat Creek Trib @ U/S CR 250 S Bridge | 38º 24.77' | -87º 19.46' |
| 66 | Pike | Flat Creek Trib @ U/S State Hwy 57 | 38º 23.94' | -87º 18.73' |
| 67 | Gibson | Hurricane Creek @ CR 00 | 38º 21.42' | -87º 20.59' |
| 68 | Gibson | Hurricane Creek @ U/S CR 50 N | 38º 21.79' | -87º 20.77' |
| 69 | Gibson | Hurricane Creek @ U/S CR 150 N | 38º 22.66' | -87º 21.58' |
| 70 | Pike | Robinson Creek @ U/S Chandler Rd Bridge | 38º 25.31' | -87º 21.64' |
| 71 | Pike | Robinson Creek @ U/S CR 200 | 38º 23.53' | -87º 21.69' |
| 72 | Gibson | Keg Creek @ CR 250 S | 38º 19.09' | -87º 21.63' |
| 73 | Gibson | East Fork Keg Creek @ U/S CR 125 S Bridge | 38º 20.24' | -87º 22.99' |
| 74 | Gibson | Patoka River @ CR 850 E | 38º 22.65' | -87º 24.44' |
| 75 | Gibson | Lost Creek @ U/S CR 50 N | 38º 21.8' | -87º 26.47' |
| 76 | Gibson | Patoka River @ Old Petersburg Rd | 38º 23.56' | -87º 32.89' |
| 77 | Gibson | Patoka River @ CR 350 N | 38º 23.85' | -87º 36.12' |
| 78 | Gibson | Patoka River @ S of 350 N | 38º 23.93' | -87º 42.7' |
| 79 | Spencer | Hunley Creek @ U/S CR 600 E Bridge | 38º 11.6' | -86º 56.51' |
| 80 | Dubois | Short Creek @ CR 500 W | 38º 15.67' | -86º 59.9' |
| 81 | Dubois | Patoka River @ US 231 | 38º 21.25' | -86º 56.61' |
| 82 | Dubois | Rock Creek @ U/S CR 900 S Bridge | 38º 17.53' | -87º 4.11' |
| 83 | Pike | Mill Creek @ U/S CR 450 E Bridge | 38º 22.16' | -87º 11.24' |
| 84 | Pike | Houchin Ditch Trib @ U/S CR 900 | 38º 17.39' | -87º 11.45' |
| 85 | Pike | South Fork Patoka River Trib @ U/S CR 300 E | 38º 18.68' | -87º 13.17' |
| 86 | Pike | South Fork Patoka River Trib @ U/S CR 875 S | 38º 17.57' | -87º 13.44' |
| 87 | Gibson | Wabash-Erie Canal @ U/S CR 1050 E | 38º 21.7' | -87º 22.24' |

**Supplemental materials Appendix b.** Site locations sampled during an investigation of the Big Oaks National Wildlife Refuge for fish and crayfish assemblage.  Site numbers correspond to Table 1, 3, and 4 and are shown in Figures 2.

**Supplemental materials Appendix c.** List of fish collection locations for an investigation of Muscatatuck National Wildlife Refuge. Site numbers correspond to Tables 1, 3 and 4 and sites shown in Figure 3.

| Site | County | Waterbody | Latitude | Longitude |
| --- | --- | --- | --- | --- |
| 1 | Jennings | Lake Linda @ CR 475 S bridge | 38º 54.95' | -85º 47.47' |
| 2 | Jackson | Moss Lake @ CR 475 S dam | 38º 55.11' | -85º 49.49' |
| 3 | Jackson | Moist Soil Unit South of Moss Lake | 38º 56.33' | -85º 49.56' |
| 4 | Jackson | Stansfield Lake @ US 31 bridge | 38º 56.02' | -85º 47.99' |
| 5 | Jackson | Mutton Creek d/s SR 50 bridge | 38º 57.82' | -85º 49.17' |
| 6 | Jackson | Vernon Fork Muscatatuck River @ US 31 bridge | 38º 54.38' | -85º 49.29' |
| 7 | Jackson | Richart Lake Outlet d/s CR 1300 S bridge | 38º 57.29' | -85º 47.95' |
| 8 | Jennings | Richart Lake Tributary d/s CR 900 W bridge | 38º 58.18' | -85º 46.66' |
| 9 | Jennings | Unnamed Tributary d/s CR 900 W bridge | 38º 55.82' | -85º 46.64' |
| 10 | Jennings | Unnamed Trib Storm Creek Ditch @ CR 1300 E | 38º 55.57' | -85º 47.81' |
| 11 | Jennings | Unnamed Trib @ D/S CR 900 W | 38º 58.01' | -85º 47.92' |
| 12 | Jennings | Unnamed Trib Storm Creek Ditch @ CR 1300 E | 38º 56.44' | -85º 49.21' |
| 13 | Jackson | Storm Creek Ditch @ U/S US 50 Bridge | 38º 55.88' | -85º 50' |
| 14 | Jackson | Mutton Creek Original Channel @ CR 400 N | 38º 53.32' | -85º 51.1' |
| 15 | Jackson | Sandy Branch @ D/S US 31 | 38º 54.66' | -85º 43.82' |
| 16 | Jackson | Stanfield Lake Outlet @ CR 1300 E | 38º 56.02' | -85º 47.99' |
| 17 | Jackson | Storm Creek Ditch @ CR 400 N | 38º 56.44' | -85º 48.35' |
| 18 | Jackson | Unnamed Trib Moss Lake @ E CR 400 N | 38º 56.42' | -85º 49.76' |
| 19 | Jackson | Unnamed Trib of Storm Creek @ CR 1300 E | 38º 56.96' | -85º 47.84' |
| 20 | Jackson | Mutton Creek Ditch @ D/S US 50 Bridge | 38º 57.82' | -85º 49.17' |
| 21 | Jackson | Pond @ Muscatatuck NWR | 38º 57.52' | -85º 47.9' |

**Supplemental materials Appendix d.** Site locations sampled for macroinvertebrates and crayfish during an investigation in the Muscatatuck National Wildlife Refuge.  Site numbers correspond to Tables 3-4 and sites shown in Figure 3.
